# Multiple steady statehood: the roles of productive and extractive capacities

**DOI:** 10.1007/s10887-021-09188-9

**Published:** 2021-03-13

**Authors:** Nils-Petter Lagerlöf

**Affiliations:** Department of Economics, York University, Toronto, Canada

**Keywords:** Malthusian model, Statehood, Multiple steady states, N40, N43, N45, O11, O43, J11

## Abstract

This paper proposes a model of statehood, defined as elite extraction of resources from a subject population. Different from most of the existing literature, the size of the subject population evolves endogenously in a Malthusian fashion, and the elite take into account the effects on future population levels when taxing the current population. The elite can spend extracted resources by investing in productive and extractive capacities. Productive capacity increases the size of the pie, while extractive capacity makes it easier for the elite to tax it. Together—but not each on its own—these two types of investment can give rise to multiple steady-state equilibria, such that one steady state has both a higher rate of extraction, and higher population density and output, than the other steady state. The model can also account for a positive empirical relationship between land productivity and state antiquity among countries with relatively late state development.

**Supplementary Information:**

The online version contains supplementary material available at 10.1007/s10887-021-09188-9.

## Introduction

For most of its existence the human species has lived in small bands of hunters and gatherers. Organized, complex, and hierarchical social structures—what we often call *states*—are a relatively recent phenomenon. States emerged gradually from around 3500 BCE, starting in a few corners of the world, in particular Mesopotamia, China, the Nile and Indus River Valleys, Mesoamerica, and the Andes (e.g., Service, 1975, Ch. 1; Borcan et al., 2018). A few millennia earlier, these same regions were also the first to enter the Neolithic Revolution, i.e., develop agriculture.

Many have therefore hypothesized a causal link from the rise of agriculture to statehood. One proposed mechanism has been labelled the Surplus Theory. The idea is that agriculture caused, or allowed, the rise of states by raising output per unit of land, thus creating a “surplus” which could be stored, and then feed a ruling elite. By contrast, in human societies that rely on relatively low-yielding techniques to obtain food, no such elite population can be sustained, since everyone’s labor is needed for procuring food. Variations on this broad explanatory theme can be found in, e.g., Childe (1936, 1950), Allen (1997), Diamond (1997), Hibbs and Olsson (2004), Putterman (2008, Section IV), and Borcan et al. (2020).^1^

Another mechanism, proposed by Scott (2009, 2017), Mayshar et al. (2017, 2020), has been labelled the Appropriability Theory. This emphasizes the characteristics of new crops that arrived with the Neolithic Revolution, in particular cereals. These were easier to expropriate than foods obtained through gathering or horticulture, specifically tubers. In support of this theory, Mayshar et al. (2020) document that statehood did not arise earlier in locations with higher agricultural yields overall, when controlling for the relative productivity of cereals and tubers. They also make the theoretical point that the Surplus Theory is hard to reconcile with a Malthusian model. This relates to the standard Malthusian result that steady-state incomes per agent are independent of land productivity, implying that the rate of extraction chosen by the elite should also be independent of land productivity.

In this paper we propose a unified Malthusian framework that incorporates some elements of both of these theories. Decisions in this model are made by a ruler, representing an “embryonic” state, and by a continuum of subjects, whose incomes the ruler has some ability to expropriate. [The pre-existence of a ruler is not crucial. Prior to full-fledged statehood, we can think of this agent as a “chief,” or what Sahlins (1963) labelled a “big man.” This is discussed further in Sect. 3.6.] The size of the subject population evolves over time in a Malthusian fashion and depends on how much the (embryonic) ruler extracts.

The extracted resources can be used for the ruler’s own consumption, or for two types of investment. First, he can invest in public goods, or what we call productive capacity. This captures the observation that early states were often instrumental in providing, e.g., irrigation (cf. Wittfogel, 1957; Nissen & Heine, 2009) and external defense (cf. Dal Bó et al., 2016).

Second, the ruler can accumulate power, or capacity, to more easily extract resources in the future. We refer to this as investment in extractive capacity. One example of such investments could be the costly acquisition of knowledge about writing and record keeping, which have been important components of a state’s extractive apparatus (Scott, 2009, pp. 226–234; Stasavage, 2020, pp. 93–96). Another example could be the hiring of skilled administrators (Ertman, 1997, Ch. 1).

Extractive and productive capacities are complementary: expanding production is more valuable when extracting it is easy, and improving extraction is more valuable when there is more to extract. This can give rise to multiple steady-state equilibria: one has low extractive capacity, low rates of extraction, and low levels of land productivity, population density, and output; another has high extractive capacity, high rates of extraction, and high levels of productivity, population density, and output.

The way these steady states differ is a non-trivial insight. The population is denser in the very steady state where it is taxed more heavily, which is surprising given the Malthusian framework. It is the higher productive capacity in the high-extractive steady state that sustains that denser population.

Also, the higher rate of extraction does not follow trivially from a higher level of extractive capacity. Rather, the ruler extracts more to finance investment in future extractive capacity.

As in any model with multiple steady states, shocks can push the economy from one steady state to another. For example, a positive shock to extractive capacity, holding productive capacity constant, can push it from the low-extractive to the high-extractive steady state; a shock to productive capacity can cause the same type of transition, holding extractive capacity constant. In that sense, the workings of the model seem consistent with both the Appropriability and Surplus Theories.

Moreover, we show that multiplicity of steady states hinges on the ruler being able to invest in *both* extractive *and* productive capacities; removing either channel renders the steady state unique. In other words, investments in extractive and productive capacities produce richer results together than each of them can on its own.

To explore the empirical relevance of the model, we lean on the complementarity between productive and extractive capacities. This complementarity implies that land productivity should have a greater impact on state building when the return to investing in extractive capacity is higher. That return should arguably depend on how many existing states there are to copy from.

To illustrate this, we consider an extended setting with many societies, and assume that the return to investing in extractive capacity faced by each ruler is increasing with the average level of extractive capacity across all societies. We then simulate the model, and let a few societies experience a positive shock to extractive capacity at some point, which pushes these to the high-extractive steady state. This in turn raises the return to investing in extractive capacity for the remaining societies, among which those with higher land productivity transition into statehood earlier than those with lower land productivity. This generates a positive relationship between land productivity and statehood across societies with late state development, but not among those with early state development. This pattern is consistent with cross-country data for the Eurasian continent.

The rest of this paper is organized as follows. Next, Sect. 2 discusses some of the existing literature. Section 3 sets up the benchmark model, and arrives at its main prediction about multiplicity of steady states. Section 4 then shows how this result falls apart when dropping investment in either extractive or productive capacities. Section 5 presents a simulation and some empirical evidence. Section 6 ends with a concluding discussion.

## Existing literature

This paper seeks to contribute to a strand of the economics literature studying early state development. One reason this topic matters to economists is that there seems to be long-lasting effects from early statehood on modern development. For example, Borcan et al. (2018) document that countries with very early and very late statehood tend to have lower GDP/capita levels than those with states of intermediate age. Other studies using earlier installments of the same state antiquity data (e.g., Bockstette et al., 2002; Chanda & Putterman, 2007; Chanda et al., 2014) find a mostly positive relationship. There are also some interesting correlations between early statehood and other modern outcome variables: Hariri (2012) documents that countries with older states are currently less democratic; Depetris-Chauvin (2016) finds links between early statehood and modern conflict in Africa. Theories linking the timing of statehood to democracy and other modern development outcomes include Lagerlöf (2016).

Empirical studies into the origins of statehood often focus on the natural environment as a deep-rooted factor. For example, Fenske (2014), Litina (2014), Depetris-Chauvin and Özak (2016) find that states emerge where ecological conditions promote trade and specialization. Heldring et al. (2019) link state development in the Fertile Crescent from 5000 BCE to shifts in rivers, which they argue induced provision of public goods.

One particularly influential theory of how the environment can induce state building is the so-called circumscription theory by Carneiro (1970), which holds that states tend to emerge where fertile lands are geographically delimited, e.g., by mountains. Recent research has found support for this theory. Schönholzer (2019) documents that states form at locations with locally high agricultural productivity, surrounded by areas with lower productivity. Looking at data from ancient Egypt, Mayoral and Olsson (2020) find that changes over time in the degree of circumscription—defined as the productivity gap between the taxable and non-taxable activity, and induced by variation in rainfall—seems to impact state stability. In our model, we may think of the parameters guiding the accumulation of extractive capacity as factors encompassing the degree of environmental circumscription.

Theories on the emergence of states also often focus on the environment. For example, Dal Bó et al. (2016) and Schönholzer (2019) present models where land productivity, and the degree of geographical circumscription, are drivers of state formation.^2^ Different from these models our setting is Malthusian, allowing us to study population density as an endogenous outcome.

Using a Malthusian framework should also help address some of the critique against theories linking land productivity to state formation, or what we here label the Surplus Theory. As discussed in Sect. 1, Mayshar et al. (2020) argue that such theories are hard to reconcile with Malthusian population dynamics. This poses a conundrum, given the broad consensus about the relevance of the Malthusian model for preindustrial development (see, e.g., Galor, 2010; Ashraf & Galor 2011). In the Malthusian model presented here, land productivity can indeed affect state building. This hinges on extractive capacity being endogenous: when closing down this channel agricultural productivity no longer has any effect on the rate of extraction, similar to the results of Mayshar et al. (2020, Online Appendices B); see Sect. 4.1 below. Our empirical findings suggest that endogenous extractive capacity may be most relevant when state building is done by copying and learning from existing states. This does not contradict that earlier state building could be better understood from a framework where extractive capacity is exogenous and a function of crop composition, as argued by Mayshar et al. (2020).

Finally, this paper leans on a theoretical literature, starting with Besley and Persson (2009, 2011), on investment in fiscal and legal state capacities; what we here call extractive capacity corresponds closest to fiscal capacity in their jargon. Again, one difference is that we use a Malthusian setting, where population density is endogenous.^3^

## The model

Consider a world with two classes: subjects and what we for simplicity call a “ruler.” The term ruler, and many model assumptions, are discussed further in Sect. 3.6.

The subjects live in overlapping generations for two periods: as passive children and active adults. In the adult phase of life, a subject works, pays taxes, and produces offspring. This means that the size of the subject population evolves endogenously over time, as a function of the ruler’s extraction rate.

The ruler has one single offspring who replaces him in the next period. We refer to him by the singular male pronoun, but this can also be interpreted as a collective of agents (an elite, or proto-elite).^4^

The ruler decides on the rate at which subjects are taxed, denoted $\tau _{t}$. A fraction $1-z_{t}$ of the taxed (extracted) resources are lost, where $z_{t}\in (0,1]$. We refer to $z_{t}$ as *extractive capacity*. The subjects thus get a fraction $1-\tau _{t}$ of total output, the ruler gets a fraction $\tau _{t}z_{t}$, while the remainder, $\tau _{t}(1-z_{t})$, is lost. As discussed in Sect. 3.6, lost tax revenue can be interpreted as theft by a class of tax collectors.

Since the ruler’s income equals $\tau _{t}z_{t}Y_{t}$, we shall refer to $z_{t}Y_{t}$ as the ruler’s *effective tax base*.^5^

### Production

Output in period *t*, denoted $Y_{t}$, is produced with the production function

1 $$\begin{aligned} Y_{t}=(MBA_{t})^{\alpha }L_{t}^{1-\alpha }, \end{aligned}$$

where $\alpha$ is the land share of output, $L_{t}$ is the size of the subject population, *M* denotes the size of land (below normalized to one, $M=1$), and *B* and $A_{t}$ are the two different land productivity factors. We refer to $L_{t}$ as just population, but since land is normalized to unity, it also measures population density.

The factor *B* is taken as given by the ruler, and captures time-invariant factors determined by geography, such as the caloric content of the crops that can be grown in a particular environment. By contrast, $A_{t}$ depends on productivity-enhancing investment undertaken by the ruler, representing public goods such as irrigation systems, or knowledge. We shall refer to $A_{t}$ as *productive capacity*.^6^

### Extraction and population dynamics

Each subject earns the average product of labor, $y_{t}=Y_{t}/L_{t}=(BA_{t}/L_{t})^{\alpha }$, which is taxed at rate $\tau _{t}\in [0,1]$. Each subject’s income after tax thus equals $(1-\tau _{t})y_{t}$.

Subjects care about consumption, $c_{t}^{S}$, and fertility, $n_{t}$, and utility is given by

2 $$\begin{aligned} U_{t}^{S}=(1-{\widetilde{\gamma }})\ln c_{t}^{S}+\widetilde{\gamma }\ln n_{t} , \end{aligned}$$

where ${\widetilde{\gamma }}\in \left( 0,1\right)$. Each subject takes her income as given and maximizes (2) subject to the budget constraint

3 $$\begin{aligned} c_{t}^{S}=(1-\tau _{t})y_{t}-qn_{t}, \end{aligned}$$

where $q>0$ is the cost per child. This gives optimal fertility as

4 $$\begin{aligned} n_{t}=\gamma (1-\tau _{t})y_{t}. \end{aligned}$$

where $\gamma \equiv {\widetilde{\gamma }}/q$. Since each subject is replaced by $n_{t}$ offspring, the subject population in the next period equals $L_{t+1}=n_{t}L_{t}$. Applying (4) and $y_{t}=Y_{t}/L_{t}$ gives

5 $$\begin{aligned} L_{t+1}=\gamma (1-\tau _{t})y_{t}L_{t}=\gamma (1-\tau _{t})Y_{t}. \end{aligned}$$

The subject population thus constitutes a capital stock to the ruler, in the sense that its size in the next period, $L_{t+1}$, decreases with the ruler’s current rate of extraction, $\tau _{t}$. Put another way, $1-\tau _{t}$ is the fraction of output that the ruler “invests” in the subject population.

### Investment in extractive capacity

Let the ruler’s investment in next period’s extractive capacity be denoted $x_{t}\ge 0$, which builds extractive capacity in the next period, $z_{t+1}$ , at a rate $\phi >0$. We let extractive capacity be bounded from above and below at levels ${\overline{z}}$ and ${\underline{z}}$, respectively, such that $0<{\underline{z}}<{\overline{z}}\le 1$ (discussed further in Sect. 3.6 below). More precisely,

6 $$\begin{aligned} z_{t+1}=\min \{{\overline{z}},{\underline{z}}+\phi x_{t}\}=\left\{ \begin{array}{lll} {\overline{z}} &{} \text {if} &{} x_{t}\ge \frac{{\overline{z}}-{\underline{z}}}{\phi }, \\ {\underline{z}}+\phi x_{t} &{} \text {if} &{} x_{t}\in \left( 0,\frac{{\overline{z}}- {\underline{z}}}{\phi }\right) , \\ {\underline{z}} &{} \text {if } &{} x_{t}=0. \end{array} \right. \end{aligned}$$

The parameter $\phi$ is a measure of how easy extractive capacity is to build. For now this is treated as exogenous. In Sect. 5 we are going to interpret $\phi$ as a function of extractive capacity among other societies, the idea being that state building is often done by copying existing states.^7^

### Investment in productive capacity

Consider next investment in productive capacity. We let the cost of $A_{t+1}$ in terms of period-*t* consumption be $\eta A_{t+1}^{\sigma }$, where $\eta >0$ and $\sigma >1$. Assuming $\sigma >1$ ensures that output and population converge to constant non-growing levels. The ruler’s budget constraint can now be written

7 $$\begin{aligned} c_{t}^{R}=\tau _{t}z_{t}Y_{t}-\eta A_{t+1}^{\sigma }-x_{t}, \end{aligned}$$

where $c_{t}^{R}$ is the ruler’s consumption.

### Utility

The ruler’s preferences are defined over $c_{t}^{R}$ and the total effective tax base in the next period, $z_{t+1}Y_{t+1}$, with utility function

8 $$\begin{aligned} U_{t}^{R}=(1-\beta )\ln \left( c_{t}^{R}\right) +\beta \ln (z_{t+1}Y_{t+1}) , \end{aligned}$$

where $$\beta \in \left( 0,1\right)$$.^8^

### Discussion

Before we set up the ruler’s maximization problem, it is helpful to scrutinize some of the (implicit and explicit) assumptions in the set-up so far.

#### Minimum extractive capacity

As mentioned, we assume upper and lower bounds for extractive capacity, denoted ${\overline{z}}$ and ${\underline{z}}$, respectively. The upper bound is not critical and can be set to one, ${\overline{z}}=1$. The assumption that ${\underline{z}}>0$ is more important. If ${\underline{z}}=0$, then the economy would under certain conditions converge to a steady state with zero population and output, a special case of what we will later call a low-extractive steady state. Intuitively, in that steady state the ruler would have no extractive capacity, and thus lack tax revenue with which to invest in productive capacity, which is necessary for production, and thus for the population to reproduce. Assuming a minimum level of extractive capacity ensures that this steady state has positive population.

There are other ways to avoid the outcome with a vanishing population. For example, one can impose an exogenous lower bound for productive capacity instead.^9^ However, that type of model would be mechanically similar to the one set up here, the main difference being that a non-negativity constraint on investment in productive capacity would replace that for extractive capacity in the current set-up.

#### Egalitarianism and the assumed pre-existence of a ruler

The model presumes that a so-called ruler exists, which might ostensibly contradict the idea of an egalitarian social structure from which statehood emerges. Again, this is mostly for simplicity and clarity, and not completely at odds with the stylized facts pertaining to many pre-state societies.

First of all, the ruler does not need to be *richer* than other agents. The Online Appendices shows that the ruler’s steady-state income can be lower than, or equal to, that of his subjects, if ${\underline{z}}$ is sufficiently small. What distinguishes the ruler from the subjects is not his income, but rather that he chooses taxes and invests in extractive and productive capacities.

Second, in any economic model where variation in statehood is the endogenous result of a choice, that choice needs to be vested with some agent, whether we call that agent a “ruler” or something else, and whatever the exact choice is. When interpreting the model, we may think of the decision maker more abstractly, standing in for various mechanisms through which pre-state societies solve collective-action problems, e.g., processes involving collaboration and negotiation.

Third, the conjectured presence of *some* type of ruler may in fact hold true for many quasi-egalitarian and pre-agrarian societies. It is common to categorize the political organization of human societies on a gradient from egalitarian bands, via more unequal tribes and chiefdoms, to fully fledged and highly hierarchical states (Flannery, 1972; Service, 1975; Diamond, 1997). In our model, equilibrium outcomes with low extractive capacity could at least correspond to chiefdoms.

Moreover, some societies at the earlier political stages have also been described as having embryonic rulers, tasked with rudimentary forms of public goods provision. Read (1959) coined the term “big man” for such leader figures among pre-state societies in New Guinea. Sahlins (1963) used the same term to contrast leader figures in Melanesia to those in more politically advanced Polynesian chiefdoms; see Lindstrom (1981) for other terminology used in the literature, such as “head man” and “center man.” Different from rulers of states, these leaders were typically not bestowed their powers through office or inheritance, but rather personal traits (Service, 1975, pp. 49–53). This may correspond to ${\underline{z}}$ in our model, applying when the preceding ruler did not invest in extractive capacity (by setting $x_{t}=0$).

#### Defense against external predators

The variable $A_{t}$ is referred to as productive capacity. This may also include defensive (or protective) capacity. Specifically, we could let some fraction of the output be stolen by external predators, and allow the ruler to undertake costly investments to limit that fraction. That setting is explored in the Online Appendices, and shown to boil down to the same one presented here. The main difference is that some of the variables that we here treat as exogenous, such as $\eta$ and $\sigma$, in that setting become functions of the “deep” parameters characterizing the costs of investing in productive and defensive capacities, respectively.

One insight from that model set-up is that land that is less costly to protect corresponds to more productive land in the current setting (i.e., a higher *B*). Intuitively, resources not needed for protection can be invested in productive capacity instead, which translates to more output at a given level of total investment in defensive and productive capacities. In that sense, we can think of *B* as a measure not only of land productivity, but also of how well protected output is.^10^

#### Tax collectors

We have conceptualized extractive capacity in this model as the fraction of the taxes collected that end up with the ruler, rather than being lost in the process of collecting them.

In order to not restrict ourselves to one single interpretation, we have not explicitly modelled *how* those tax revenues are lost. The Online Appendices proposes one way to capture that process more explicitly by introducing a new class of agents, called tax collectors. These can run off with the taxes they collect, and the ruler can invest in capacity to retrieve (some of) those lost revenues. The upshot is a model producing the same functional form for accumulation of extractive capacity as that in (6), but with ${\overline{z}}$, ${\underline{z}}$, and $\phi$ being functions of “deep” model parameters.

#### Alternative ways to model extractive capacity

There are other ways to model extractive capacity. We can let the ruler face a cost of levying taxes, incurred in the same period they are levied. Then extractive capacity, $z_{t}$, could be a variable characterizing that cost function, such that a higher $z_{t}$ implies a lower cost of tax collection. This formulation resembles that of Mayshar et al. (2020, Online Appendices B).

Specifically, let the cost of levying a tax rate of $\tau _{t}$ on total output $Y_{t}$ equal $C\left( \tau _{t},z_{t}\right) Y_{t}$, where $C\left( \tau _{t},z_{t}\right)$ is increasing in the tax rate, $\tau _{t}$, and decreasing in $z_{t}$. Then the ruler’s budget constraint, corresponding to that in (7), becomes

9 $$\begin{aligned} c_{t}^{R}=\left[ \tau _{t}-C\left( \tau _{t},z_{t}\right) \right] Y_{t}-\eta A_{t+1}^{\sigma }-x_{t}. \end{aligned}$$

Our setting can be seen as a special case of this formulation, where $C\left( \tau _{t},z_{t}\right) =\tau _{t}(1-z_{t})$, which makes (9 ) identical to (7). Similarly, what we can call the *net* tax (or extraction) rate, $\tau _{t}-C\left( \tau _{t},z_{t}\right)$, then equals just $z_{t}\tau _{t}$, which corresponds more closely to the variable used to measure statehood in Mayshar et al. (2020, Online Appendices B). In our benchmark model both $\tau _{t}$ and $z_{t}$ are endogenous, while they treat the latter as exogenous.

### The ruler’s optimization problem

We are now ready to set up the ruler’s optimization problem. Recall that he chooses $\tau _{t}$, $x_{t}$, and $A_{t+1}$ to maximize (8), subject to (5), (6), (7), (1) forwarded one period, and a non-negativity constraint on $x_{t}$. More compactly, the problem can be written as follows:

10 $$\begin{aligned} \max _{\tau _{t},x_{t},A_{t+1}}(1-\beta )\ln \left( c_{t}^{R}\right) +\beta \ln (z_{t+1}Y_{t+1}), \end{aligned}$$

subject to

11 $$\begin{aligned} \begin{array}{l} x_{t}\ge 0, \\ z_{t+1}=\min \{{\overline{z}},{\underline{z}}+\phi x_{t}\}, \\ c_{t}^{R}=\tau _{t}z_{t}Y_{t}-\eta A_{t+1}^{\sigma }-x_{t}, \\ Y_{t+1}=(BA_{t+1})^{\alpha }L_{t+1}^{1-\alpha }, \\ L_{t+1}=\gamma (1-\tau _{t})Y_{t}. \end{array} \end{aligned}$$

We refer to this as the benchmark model. Its results can be understood from three different trade-offs that the ruler faces. First, higher investment in productive capacity, $A_{t+1}$, generates a larger tax base in the next period (higher $Y_{t+1}$), at the cost of less consumption for the ruler today (lower $c_{t}^{R}$).

Second, a higher extraction rate, $\tau _{t}$, gives higher income and consumption today (by raising more tax revenue, $\tau _{t}z_{t}Y_{t}$); this comes at the cost of a smaller future tax base (lower $Y_{t+1}$), in turn due to the Malthusian way in which more extraction reduces the future population size ($L_{t+1}$).

Third, investment in future extractive capacity, $z_{t+1}$, is costly in terms of current consumption.

Due to the assumed linear functional form, and the upper and lower bounds on $z_{t+1}$, this last trade-off can be seen to generate corner solutions: by setting $x_{t}=0$, and thus $z_{t+1}={\underline{z}}$, the ruler invests nothing in extractive capacity, keeping it at its minimum level; by setting $x_{t}=({\overline{z}}-{\underline{z}})/\phi$, and thus $z_{t+1}={\overline{z}}$, the ruler chooses maximum extractive capacity.

The ruler’s investment in future extractive capacity depends on his current effective tax base, $z_{t}Y_{t}$. If this is small, then a marginal increase in $\tau _{t}$ generates relatively little revenue, thus making it costly to finance investment in extractive capacity. If the effective tax base is small enough it is optimal to set $x_{t}=0$; if it is sufficiently large, then it is optimal to set $x_{t}=({\overline{z}}-{\underline{z}})/\phi$. In that sense, a currently strong and rich state is more likely to remain strong also in the next period. The next section derives explicit expressions for the ruler’s choice variables as functions of the effective tax base and exogenous parameters (with details deferred to Sect. 1 of the Appendices).

### The ruler’s optimal choices

Let ${\underline{X}}$ and ${\overline{X}}$ denote the thresholds for $z_{t}Y_{t}$ , above and below which the two constraints on $z_{t+1}$ in (6) bind. That is, $x_{t}=0$ and $z_{t+1}={\underline{z}}$ if $z_{t}Y_{t}\le {\underline{X}}$; and $x_{t}=({\overline{z}}-{\underline{z}})/\phi$ and $z_{t+1}={\overline{z}}$ if $z_{t}Y_{t}\ge {\overline{X}}$. A weak ruler, with a low effective tax base ($z_{t}Y_{t}\le {\underline{X}}$), finds current extraction costly, making it optimal not to build any future extractive capacity, thus preserving the weak state. A strong ruler, with a large effective tax base ($z_{t}Y_{t}\ge {\overline{X}}$), finds it easy to extract resources, and chooses to maintain a strong state by investing enough to keep extractive capacity to its maximum, ${\overline{z}}$.

As shown in Sect. 1 of the Appendices, these thresholds are given by

12 $$\begin{aligned} {\overline{X}}=\frac{1}{\phi }\left[ {\overline{z}}\left( \frac{\beta \sigma (1-\alpha )+\sigma +\alpha \beta }{\beta \sigma }\right) -{\underline{z}} \right] , \end{aligned}$$

and

13 $$\begin{aligned} {\underline{X}}=\frac{1}{\phi }\left( \frac{\sigma (1-\alpha \beta )+\alpha \beta }{\beta \sigma }\right) {\underline{z}}. \end{aligned}$$

It is straightforward to show that $0<{\underline{X}}<{\overline{X}}$ follows from $0<{\underline{z}}<{\overline{z}}$.

The ruler’s choices thus depend on how the effective tax base falls relative to these thresholds. Consider first how the ruler sets the rate of extraction. Section 1 of the Appendices shows that the ruler’s optimal extraction rate can be written:

14 $$\begin{aligned} \tau _{t}=\left\{ \begin{array}{lll} 1-\left[ \frac{\beta \sigma (1-\alpha )}{\sigma (1-\alpha \beta )+\alpha \beta }\right] \left[ 1-\left( \frac{{\overline{z}}-{\underline{z}}}{\phi } \right) \frac{1}{z_{t}Y_{t}}\right] &{} \text {if} &{} z_{t}Y_{t}\ge {\overline{X}} , \\ 1-\left( \frac{\beta \sigma (1-\alpha )}{\beta \sigma (1-\alpha )+\sigma +\alpha \beta }\right) \left( 1+\frac{{\underline{z}}}{\phi z_{t}Y_{t}}\right) &{} \text {if} &{} z_{t}Y_{t}\in \left[ {\underline{X}},{\overline{X}}\right] , \\ 1-\left[ \frac{\beta \sigma (1-\alpha )}{\sigma (1-\alpha \beta )+\alpha \beta }\right] =\frac{\sigma (1-\beta )+\alpha \beta }{\sigma (1-\alpha \beta )+\alpha \beta } &{} \text {if } &{} z_{t}Y_{t}\le {\underline{X}}. \end{array} \right. \end{aligned}$$

It can be see from (14) that the relationship between $\tau _{t}\,$ and $z_{t}Y_{t}$ is inversely U-shaped. First, $\tau _{t}$ is constant for $z_{t}Y_{t}\le {\underline{X}}$, i.e., when investment in extractive capacity is not operative. This constant rate is the same as in the corresponding model without any investment in extractive capacity (see Sect. 4.1).

We also see that $\tau _{t}$ is increasing in $z_{t}Y_{t}$ for $z_{t}Y_{t}\in \left[ {\underline{X}},{\overline{X}}\right]$. Over this interval, rulers respond to marginal increases in the effective tax base ($z_{t}Y_{t}$) by extracting more resources, in order to fund more investment in future extractive capacity. Finally, we see that $\tau _{t}$ decreases with $z_{t}Y_{t}$ for $z_{t}Y_{t}\ge {\overline{X}}$. Intuitively, the cost of maintaining maximum extractive capacity falls relative to income as the effective tax base grows.

As $z_{t}Y_{t}$ approaches infinity, $\tau _{t}$ approaches the same level as when $z_{t}Y_{t}\le {\underline{X}}$. However, for any finite level of $z_{t}Y_{t}$, the extraction rate is always higher when the ruler invests the maximum amount in future extractive capacity ($z_{t}Y_{t}\ge {\overline{X}}$ and $z_{t+1}={\overline{z}}$) than when he invests the minimum amount ($z_{t}Y_{t}\le {\underline{X}}$ and $z_{t+1}={\underline{z}}$). That is, the top row of (14) is always greater than the bottom row, for finite $z_{t}Y_{t}$. This means that any steady state with maximum investment in extractive capacity must have a higher extraction rate than one with no such investment. Below we explore if two such steady states can coexist.

### Dynamics

Since the optimal extraction rate in (14) depends on the effective tax base, $z_{t}Y_{t}$, the dynamics of the economy are most easily described in terms of the two state variables $Y_{t}$ and $z_{t}$.

#### Dynamics of $z_{t}$

As shown in Sect. 1 of the Appendices, the ruler’s optimal choice of $z_{t+1}$ (as implied by the choice of $x_{t}$) can be written

15 $$\begin{aligned} z_{t+1}=\Phi (Y_{t},z_{t})\equiv \left\{ \begin{array}{lll} {\overline{z}} &{} \text {if} &{} z_{t}Y_{t}\ge {\overline{X}}, \\ \left( \frac{\beta \sigma }{\beta \sigma (1-\alpha )+\sigma +\alpha \beta } \right) \left[ \phi z_{t}Y_{t}+{\underline{z}}\right] &{} \text {if} &{} z_{t}Y_{t}\in \left[ {\underline{X}},{\overline{X}}\right] , \\ {\underline{z}} &{} \text {if } &{} z_{t}Y_{t}\le {\underline{X}}. \end{array} \right. \end{aligned}$$

That is, $z_{t+1}\ge {\underline{z}}$ binds when $z_{t}Y_{t}<{\underline{X}}$, and $z_{t+1}\le {\overline{z}}$ binds when $z_{t}Y_{t}>{\overline{X}}$. When these constraints are non-binding (i.e., when $z_{t}Y_{t}\in \left[ {\underline{X}},{\overline{X}}\right]$) the next period’s extractive capacity ($z_{t+1}$) increases linearly with the current period’s effective tax base ($z_{t}Y_{t}$). It is also easy to verify that the respective corner solutions coincide with the interior solution when $z_{t}Y_{t}={\underline{X}}$ and $z_{t}Y_{t}={\overline{X}}$.

#### Dynamics of $Y_{t}$

From (1) we see that $Y_{t+1}=(BA_{t+1})^{\alpha }L_{t+1}^{1-\alpha }$ , and from (5) we recall that $L_{t+1}=\gamma (1-\tau _{t})Y_{t}$. Once we have the ruler’s optimal $A_{t+1}$ and $\tau _{t}$ in terms of $z_{t}$ and $Y_{t},$ we can thus derive an expression for $Y_{t+1}$ in terms of the same state variables. Section 2 of the Appendices shows that

16 $$\begin{aligned} Y_{t+1}=\Psi (Y_{t},z_{t},B)\equiv \left\{ \begin{array}{lll} \kappa DB^{\alpha }z_{t}^{\alpha -1}\left[ \phi z_{t}Y_{t}+{\underline{z}}- {\overline{z}}\right] ^{\rho } &{} \text {if} &{} z_{t}Y_{t}\ge {\overline{X}}\text {, } \\ DB^{\alpha }z_{t}^{\alpha -1}\left[ \phi z_{t}Y_{t}+{\underline{z}}\right] ^{\rho } &{} \text {if} &{} z_{t}Y_{t}\in \left[ {\underline{X}},{\overline{X}}\right] , \\ \kappa DB^{\alpha }z_{t}^{\alpha -1}\left( \phi z_{t}Y_{t}\right) ^{\rho } &{} \text {if } &{} z_{t}Y_{t}\le {\underline{X}}, \end{array} \right. \end{aligned}$$

where $\rho =(\alpha /\sigma )+1-\alpha <1$, and where $D>0$ and $\kappa >1$ depend only on the exogenous and time-invariant variables $\alpha$, $\beta$ , $\gamma$, $\phi$, $\sigma$, and $\eta$ [see (47) and (54 ) in the Appendices], and play no role for the dynamics.

Note that $Y_{t+1}$ depends on *B*, i.e., the land productivity factor that is independent of the ruler’s investment. This has interesting implications for how changes in *B* impact the dynamic configuration, as discussed below.

#### Multiple steady states

**Fig. 1:**
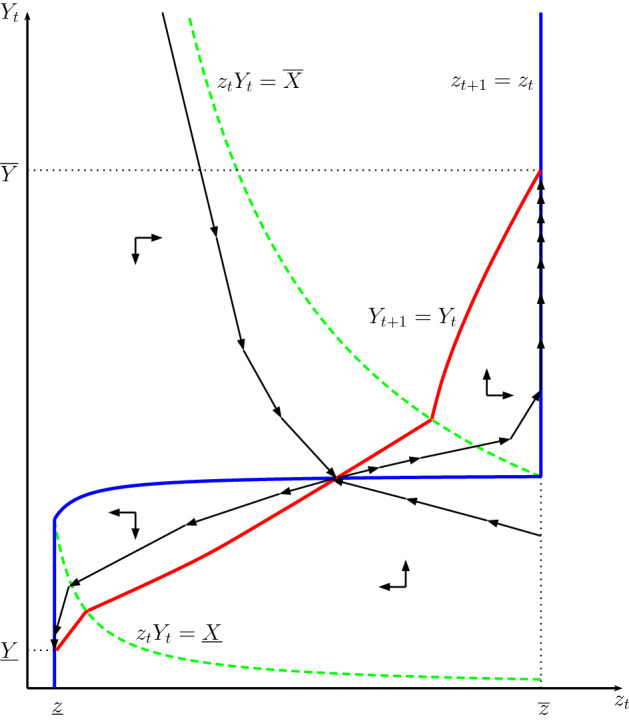
Phase diagram illustrating the dynamics. The loci along which $z_{t}$ and $Y_{t}$ are constant are indicated by the red and blue solid curves. The green dashed curves indicate the loci above and below which the constraints $z_{t+1} \le {\overline{z}}$ and $z_{t+1} \ge {\underline{z}}$ bind. In this configuration, there exist two stable steady states (Color figure online)

Now (15) and (16) define a two-dimensional dynamical system for $z_{t}$ and $Y_{t}$, which is illustrated in the phase diagram in Fig. 1. It shows the loci along which $z_{t}$ and $Y_{t}$ are constant (derived in Sect. 3 of the Appendices), and the regions where the constraints on extractive-capacity investment bind: $z_{t+1}\ge {\underline{z}}$ binds when $z_{t}Y_{t}<{\underline{X}}$, and $z_{t+1}\le {\overline{z}}$ binds when $z_{t}Y_{t}>{\overline{X}}$.

Generally, the configuration depends on exogenous variables, in particular *B*. Figure 1 illustrates a case where there are two locally stable steady-state equilibria, and one unstable. (Exact conditions for this type of configuration are stated in Proposition 1 below.) One stable steady-state equilibrium can be labelled a *low-extractive* steady state. Here the ruler undertakes no investment in extractive capacity, so $z_{t}={\underline{z}}$, and output can be written

17 $$\begin{aligned} {\underline{Y}}=\left[ \kappa DB^{\alpha }{\underline{z}}^{\alpha -1}\left( \phi {\underline{z}}\right) ^{\rho }\right] ^{\frac{1}{1-\rho }}, \end{aligned}$$

which is illustrated in Fig. 1, and derived by setting $Y_{t+1}=Y_{t}={\underline{Y}}$ and $z_{t}={\underline{z}}$ in the bottom row of ( 16). The associated extraction rate, which we can denote ${\underline{\tau }}$, is given by the bottom row of (14), i.e., ${\underline{\tau }} =[\sigma (1-\beta )+\alpha \beta ]/[\sigma (1-\alpha \beta )+\alpha \beta ]$ . Population is given by (5) as ${\underline{L}}=\gamma (1-\underline{ \tau }){\underline{Y}}$.

The other stable steady state, at which $z_{t}={\overline{z}}$, can be labelled the *high-extractive* steady state. Here output equals ${\overline{Y}}$, defined from ${\overline{Y}}=\kappa DB^{\alpha }{\overline{z}} ^{\alpha -1}\left[ \phi {\overline{z}}{\overline{Y}}+{\underline{z}}-{\overline{z}} \right] ^{\rho }$; cf. the top row of (16). The extraction rate in this steady state, ${\overline{\tau }}$, is given by the top row of (14 ), setting $z_{t}Y_{t}={\overline{z}}{\overline{Y}}$. From (5), population can be written ${\overline{L}}=\gamma (1-{\overline{\tau }}){\overline{Y}}$.

A saddle path separates the phase diagram into two basins of attraction, each associated with one of the two steady states.^11^ An economy starting off above the saddle path (i.e., with a large initial effective tax base, $z_{0}Y_{0}$) will converge over time to the high-extractive steady state. An economy starting off below the saddle path converges to the low-extractive steady state.

A trajectory leading to the high-extractive steady state eventually enters a region where $z_{t}Y_{t}>{\overline{X}}$, at which point the upper bound on extractive capacity investment starts to bind. From there, $z_{t}$ stays constant at ${\overline{z}}$, while $Y_{t}$ continues to grow, stabilizing at ${\overline{Y}}$, as illustrated in Fig. 1. Similarly, a trajectory leading to the low-extractive steady state eventually enters a region where $z_{t}Y_{t}<{\underline{X}}$, after which $z_{t}$ stays constant at ${\underline{z}}$, while $Y_{t}$ declines, approaching ${\underline{Y}}$.

We can also compare levels of population, output, extractive capacity, and rates of extraction in the two steady states. This is a nontrivial exercise, since these are all endogenous and jointly determined. The following proposition summarizes these results, and provides conditions for the existence and uniqueness of each steady state, respectively.

##### **Proposition 1**

*Consider the model with investment in both productive and extractive capacities, as described by* (10) *and* (11). *In this model, there exist* ${\widehat{B}}>0$ *and* $\widehat{{\widehat{B}}}>0$, *such that*:

- *If, and only if*, $B<{\widehat{B}}$ *does there exist a low-extractive steady state*, $({\underline{z}},{\underline{Y}})$, *such that* ${\underline{z}}{\underline{Y}}<{\underline{X}}$.
- *If, and only if*, $B>\widehat{{\widehat{B}}}$ *does there exist a high-extractive steady state*, $({\overline{z}},{\overline{Y}})$, *such that* ${\overline{z}}{\overline{Y}}>{\overline{X}}$.
- *For* ${\underline{z}}$ *small enough, it holds that* $\widehat{ {\widehat{B}}}<{\widehat{B}}$. *That is, the low- and the high-extractive steady states coexist for* $B\in (\widehat{{\widehat{B}}},{\widehat{B}})$.
- *Assume that* $B\in (\widehat{{\widehat{B}}},{\widehat{B}})$, *so that both steady states exist. Then the following holds*:
  (i) *The low-extractive steady state has a lower extraction rate than the high-extractive steady state, i.e.*, ${\underline{\tau }}< {\overline{\tau }}$;
  (ii) *The low-extractive steady state has lower output than the high-extractive steady state, i.e.,* ${\underline{Y}}<{\overline{Y}}$;
  (iii) *The low-extractive steady state has lower population than the high-extractive steady state, i.e.*, ${\underline{L}}<{\overline{L}}$.

All proofs are in Sect. 5 of the Appendices.

The possibility of multiple steady states is quite intuitive, and has to do with how current extraction affects future extraction. A larger initial level of the effective tax base—i.e., a larger $z_{t}Y_{t}$—induces the ruler to invest more in both $z_{t+1}$ and $Y_{t+1}$, leading to a larger effective tax base in the next period. This can sustain high levels of extractive and productive capacities across generations of rulers. As we shall see in Sect. 4 below, investment in productive and extractive capacities are both needed for multiplicity of steady-state equilibria to arise.

The claims in part (d) in Proposition 1, comparing the properties of these steady states, are far less obvious.

For example, part (d) (iii) states that the high-extractive steady state has larger population (density) than the low-extractive one (${\underline{L}}< {\overline{L}}$). This may seem counter-intuitive, since a higher rate of extraction [see (d) (i)] would imply a smaller population for a given level of output; to see this one can impose steady state on (5). The result still holds because output is higher in the high-extractive steady state [see (d) (ii)], in turn due to higher investment in productive capacity, which is sustained by the ruler’s larger tax revenues.

Part (d) (i) of Proposition 1 is not obvious either (despite the ostensibly self-explanatory labels). We gleaned some of the intuition from ( 14). It is not merely about higher extractive capacity inducing a higher rate of extraction. In fact, the rate of extraction in the low-extractive steady state (${\underline{\tau }}$) is independent of the exogenously given minimum level of extractive capacity (${\underline{z}}$).^12^ In other words, small changes in extractive capacity do not affect the rate of extraction, as long as the economy is not pushed out of the low-extractive steady state. Rather, the result refers specifically to a steady-state comparison. In the high-extractive steady state the ruler chooses a higher rate of extraction to finance investment in future extractive capacity, which is worthwhile precisely because of the large effective tax base in that steady state.

**Shocks to** $z_{t}$ **or** $Y_{t}$ As explained above, given a configuration with multiple steady states, such as that in Fig. 1, the economy converges over time to one of the stable steady-state equilibria. Which one it converges to depends on its initial position relative to the saddle-path trajectory leading to the unstable steady state.

This means that an economy can transition from the low-extractive to the high-extractive steady state in the wake of a one-period shock to either extractive capacity ($z_{t}$), or output ($Y_{t}$), or a combination of the two. Intuitively, the shock raises the ruler’s effective tax base in period *t*, inducing him to invest more in productive and/or extractive capacity, possibly putting the economy on a trajectory leading to the high-extractive steady state. For this to happen, the shock must push ($z_{t},Y_{t}$) above the threshold saddle path, into the basin of attraction of the high-extractive steady state.

A transition due to a shock to output would be consistent with the Surplus Theory, and could perhaps be interpreted as the result of temporary climatic variations, and/or a temporary phase of good harvests. A transition due to a shock to extractive capacity relates conceptually to the Appropriability Theory.

**Exogenous changes to** *B* Above we considered shocks to extractive capacity ($z_{t}$) or output ($Y_{t}$). We can also analyze exogenous increases in the geographically determined land productivity factor, *B*. As shown in Sect. 3 of the Appendices, this shifts up the ($Y_{t+1}=Y_{t}$)-locus, thus raising output in the low-extractive steady state; note from (17) that ${\underline{Y}}$ is increasing in *B*. It also expands the basin of attraction for the high-extractive steady state. At some point the low-extractive steady state ceases to exist. Intuitively, a rise in *B* implies more output, which in turn can be used to accumulate both productive and extractive capacities.

Changes in *B* need not be interpreted as shocks. Very gradual increases in *B* would have small effects at first, but eventually lead to rapid changes in $z_{t}$ and $Y_{t}$, as the dynamic configuration changes and the high-extractive steady state becomes the unique steady state (i.e., when *B* exceeds ${\widehat{B}}$). The economy can thus initially change slowly in response to improvements in *B*, and then go through a rapid spurt in extractive capacity and output, stabilizing at ${\overline{z}}$ and ${\overline{Y}}$, respectively. From there, output expands more slowly again (as ${\overline{Y}}$ is increasing in *B*).

## Closing down channels

In the benchmark model the ruler could invest in both extractive and productive capacities. To see why this matters, we next consider what happens when we close down either of these channels.

### Closing down investment in extractive capacity

To remove investment in extractive capacity from the model, we ignore (6), setting $x_{t}=0$, and let $z_{t}$ equal some exogenous constant, here denoted ${\widetilde{z}}\in (0,1]$. In this setting, an increase in ${\widetilde{z}}$ represents a rise in extractive capacity independent of any actions taken by the ruler, conceptually similar to Mayshar et al. (2020, Online Appendices B), who treat extractive capacity as exogenous.

The ruler’s optimization problem now becomes:

18 $$\begin{aligned} \max _{\tau _{t},A_{t+1}}(1-\beta )\ln \left( c_{t}^{R}\right) +\beta \ln ( {\widetilde{z}}Y_{t+1}), \end{aligned}$$

subject to

19 $$\begin{aligned} \begin{array}{l} c_{t}^{R}=\tau _{t}{\widetilde{z}}Y_{t}-\eta A_{t+1}^{\sigma }, \\ Y_{t+1}=(BA_{t+1})^{\alpha }L_{t+1}^{1-\alpha }, \\ L_{t+1}=\gamma (1-\tau _{t})Y_{t}. \end{array} \end{aligned}$$

The solution to this model resembles that analyzed in the previous section in the case when the non-negativity constraint on $x_{t}$ was binding ($x_{t}=0$); see Sect. 1 of the Appendices for details. The dynamics of output becomes

20 $$\begin{aligned} Y_{t+1}=GY_{t}^{\rho }, \end{aligned}$$

where (recall) $\rho =(\alpha /\sigma )+1-\alpha <1$, and where *G* depends on exogenous parameters and is increasing in both agricultural productivity ( *B*), and extractive capacity (${\widetilde{z}}$); see (60) in the Appendices. The following proposition summarizes the main results in this setting.

#### **Proposition 2**

*Consider the model without investment in extractive capacity, as described by* (18) *and* (19). *In this model, there exists a unique (non-zero) steady-state equilibrium where the following holds: extractive capacity equals its exogenous level*, ${\widetilde{z}}$; *output equals* ${\widetilde{Y}}=G^{1/(1-\rho )}$; *and the rate of extraction equals*

21 $$\begin{aligned} {\widetilde{\tau }}=\frac{\sigma (1-\beta )+\alpha \beta }{\sigma (1-\alpha \beta )+\alpha \beta }. \end{aligned}$$

Thus, taking investment in extractive capacity out of the model rules out multiplicity of steady states. It can be seen that ${\widetilde{Y}}$ is increasing in both *B* and ${\widetilde{z}}$ (since *G* is), so we do get the expected predictions from increases in both land productivity and extractive capacity; note that extractive capacity still affects tax revenues and thus investment in productive capacity, $A_{t+1}$.

However, optimal $\tau _{t}$ is here constant. [Indeed, the expression in (21) is the same as in the bottom row in (14), which applies to the benchmark model when $x_{t}=0$, i.e., $z_{t}Y_{t}<{\underline{X}}$.] Since the extraction rate does not depend on either *B* or ${\widetilde{z}}$, this setting cannot explain the rise of statehood as an endogenous outcome of changes in *B* and/or ${\widetilde{z}}$. In that sense, without investment in extractive capacity the model is inconsistent with both the Surplus and Appropriability Theories.^13^

### Closing down investment in productive capacity

Next we remove investment in productive capacity, setting $A_{t}=1$ in all periods, but keep investment in extractive capacity. The ruler’s budget constraint, analogous to that in (7), becomes $c_{t}^{R}=\tau _{t}z_{t}Y_{t}-x_{t}$. The expression for output in (1) becomes $Y_{t}=B^{\alpha }L_{t}^{1-\alpha }$.

The ruler’s optimization problem can now be written:

22 $$\begin{aligned} \max _{\tau _{t},x_{t}}(1-\beta )\ln \left( c_{t}^{R}\right) +\beta \ln (z_{t+1}Y_{t+1}), \end{aligned}$$

subject to

23 $$\begin{aligned} \begin{array}{l} x_{t}\ge 0, \\ z_{t+1}=\min \{{\overline{z}},{\underline{z}}+\phi x_{t}\}, \\ c_{t}^{R}=\tau _{t}z_{t}Y_{t}-x_{t}, \\ Y_{t+1}=B^{\alpha }L_{t+1}^{1-\alpha }, \\ L_{t+1}=\gamma (1-\tau _{t})Y_{t}. \end{array} \end{aligned}$$

This model coincides with that in the benchmark setting in Sect. 3 when $\sigma$ goes to infinity, i.e., when we make investment in productive capacity prohibitively expensive. Specifically, there are two thresholds for the effective tax base, ${\underline{X}}$ and ${\overline{X}}$, below and above which investment in extractive capacity is constrained to its minimum or maximum levels, respectively. Letting $\sigma$ go to infinity in (12) and (13), these thresholds can now be written

24 $$\begin{aligned} {\overline{X}}=\frac{1}{\phi }\left[ {\overline{z}}\left( \frac{\beta (1-\alpha )+1}{\beta }\right) -{\underline{z}}\right] , \end{aligned}$$

and

25 $$\begin{aligned} {\underline{X}}=\frac{(1-\alpha \beta ){\underline{z}}}{\beta \phi }. \end{aligned}$$

That is, if $z_{t}Y_{t}\le {\underline{X}}$, then $z_{t+1}={\underline{z}}$ and $x_{t}=0$; if $z_{t}Y_{t}\ge {\underline{X}}$, then $z_{t+1}={\overline{z}}$ and $x_{t}=({\overline{z}}-{\underline{z}})/\phi$.

The dynamical system describing the evolution of $z_{t}$ and $Y_{t}$ is derived in Sect. 2 of the Appendices, and can also be derived from (15) and (16) by letting $\sigma$ go to infinity, and setting $\rho =1-\alpha$. Because the resulting expressions for $z_{t+1}$ and $Y_{t+1}$ are so qualitatively similar to those in (15) and (16), we suppress these to the Appendices.

We sum up the main results in the following proposition.

#### Proposition 3

*Consider the model without investment in productive capacity, as described by* (22) *and* (23). *In this model, there exist* $$B^{*}>0$$ *and* $$B^{**}>0$$, *such that*:

- *If, and only if*, $B<B^{*}$ *does there exist a low-extractive steady state*, $({\underline{z}},{\underline{Y}})$, *such that* ${\underline{z}}{\underline{Y}}<{\underline{X}}$.
- *If, and only if*, $$B>B^{**}$$ *does there exist a high-extractive steady state*, $$({\overline{z}},{\overline{Y}})$$, *such that* $${\overline{z}}{\overline{Y}}>{\overline{X}}$$.
- $$B^{**}>B^{*}$$. *That is, the low- and the high-extractive steady states cannot coexist*.
- *If* $$B\in (B^{*},B^{**})$$, *then there exists a unique steady state*, $$(z^{\text {int}},Y^{\text {int}})$$, *such that* $$z^{\text { int}}Y^{\text {int}}\in ({\underline{X}},{\overline{X}})$$. *Furthermore, it holds that*:
  (i) *The steady-state extraction rate*, $\tau ^{\text {int}}$, *is increasing in* *B* *and* $\phi$;
  (ii) *The steady-state level of extractive capacity rate*, $z^{ \text {int}}$, *is increasing in* *B* *and* $\phi$;
  (iii) *The steady-state level of output*, $Y^{\text {int}}$, *is increasing in B and decreasing in* $\phi$;
  (iv) *The steady-state level of population density*, $L^{\text { int}}$, *does not depend on* *B* *and is decreasing in* $\phi$.

Parts (a) and (b) of Proposition 3 are consistent with the corresponding claims in Proposition 1.^14^ More (less) productive land makes the high-extractive (low-extractive) steady state more likely to exist. This is broadly consistent with the Surplus Theory.

However, part (c) of the proposition shows that multiple steady-state equilibria are not possible in this setting. If land productivity, *B*, is high enough that the high-extractive steady state exists (meaning $$B>B^{**}$$), then it is also too high for the low-extractive steady state to exist (since $$B^{**}>B^{*}$$). Intuitively, multiplicity of steady states requires strong enough feedback from current extraction to future extraction, and this feedback is weakened when rulers are not able to invest in productive capacity.

Part (d) takes this point further, by considering the case when $$B\in (B^{*},B^{**})$$. Here neither the low- or high-extractive steady state exists. Rather, the economy converges to a unique interior steady state. Interestingly, this steady state has many properties—summarized by parts (i)-(iv) of (d)—that seem inconsistent with the facts. For example, a (small) rise in land productivity, *B*, leads to a higher steady-state extraction rate and higher levels of extractive capacity, but leaves steady-state population density unchanged. Intuitively, higher land productivity raises population in the usual Malthusian way, but that is counteracted by the higher rate of extraction, and here the net effect is zero. Both those effects were present in the benchmark model, but there higher tax revenues also generated higher investments in productive capacity, which tended to increase steady-state population density. That third channel is closed down here.

Similarly, a rise in $\phi$ (which, recall, measures how easy it is to build extractive capacity) raises the steady-state extraction rate and extractive capacity, but lowers population density. This implies a * negative* association between statehood and population density, which is inconsistent with the empirical facts.

## Empirical results

The results of the model build on a complementarity between extractive and productive capacities. Intuitively, the possibility of a high-extractive steady state hinges on land productivity affecting the effective tax base and thus investment in future extractive capacity. The implication is that an increase in land productivity, *B*, is more likely to generate statehood if investments in extractive capacity are easier to undertake, i.e., if $\phi$ is large.^15^

We can explore if this holds empirically by comparing the correlation between statehood and land productivity for samples of countries with high and low $\phi$. To measure $\phi$, we may lean on a literature emphasizing how much easier elites have found it to build a state when they already have a blueprint. For example, the earliest states developed writing and bookkeeping, which were copied by elites developing states later (Scott, 2009, pp. 226–234); Stasavage, 2020, pp. 91–93). Similarly, Ertman (1997, p. 27) argues that European state building became easier at a point when rulers could hire from an existing pool of experts to serve as administrators and in the military. In a multi-society interpretation of our model, this suggests that the return to investing in extractive capacity in one society, as captured by $\phi$, could depend on the level of extractive capacity across a range of societies.

To fix ideas, suppose a group of countries have transitioned into statehood in a first wave. Since they did not have any statehood blueprints they faced a very low $\phi$, but transitioned anyhow, possibly for reasons not modelled here, and once they have transitioned they are more likely to maintain statehood moving forward (due to the multiplicity of locally stable steady states). The remaining countries, being able to draw on the state knowledge accumulated by the first wave of countries, face a higher $\phi$. The complementarity between *B* and $\phi$ should then imply that countries in the second wave transition earlier if they have higher *B*.

### A simulation example

To better understand the dynamics of a model where $\phi$ changes over time, we can first consider a simulation where in each period $\phi$ is a function of the average level of extractive capacity, $z_{t}$, across 200 societies. (For details, see Sect. 1 of the Appendices) We let these 200 societies be endowed with different levels of land productivity, *B*, which is uniformly distributed between the two thresholds discussed in Proposition 1, $\widehat{{\widehat{B}}}$ and ${\widehat{B}}$. Thus, two steady states exist initially.

All societies start off in a low-extractive steady state, with minimum extractive capacity (${\underline{z}}$), but 20 are exogenously hit by a shock at $t=40$, giving them maximum extractive capacity (${\overline{z}}$). These 20 represent early states, and have levels of *B* distributed in the same way as among the other 180. (Here we select them as every tenth society when ranked by *B*, but one can also select them randomly.) Their function in this simulation is to initiate a process through which statehood can spread: the initial rise in average $z_{t}$ raises $\phi$, in turn inducing more societies to invest in $z_{t}$, thus raising $\phi$ further, creating a self-propelling dynamic.

**Fig. 2:**
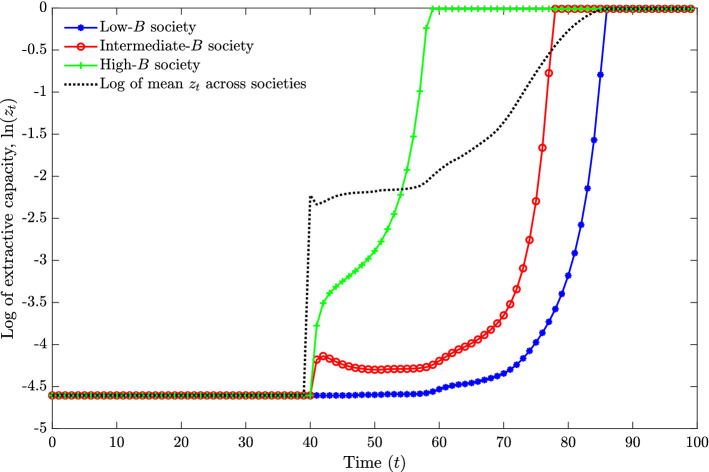
Simulated time paths over 100 periods showing log extractive capacity, $\ln (z_{t})$, for three societies in a setting where $\phi$ depends on the average level of $z_{t}$ across all societies (shown as a dotted path). These three are all among those societies not hit by a shock to extractive capacity

Figure 2 shows the simulated time paths of the log of $z_{t}$ for three societies out of the 180 not hit by the shock. A higher *B* is associated with an earlier rise in $z_{t}$, since higher land productivity induces earlier investments in $z_{t}$ when $\phi$ starts to rise; the rise in $\phi$ is in turn driven by the rise in average $z_{t}$ across the 200 societies, shown as a dotted line.

Some paths in Fig. 2 show a non-monotonic rise (hardly visible unless we log $z_{t}$), which reflects that the dynamics for a fixed $\phi$ exhibit two locally stable steady states. Depending on parameter values, not all societies need ever transition into statehood, but in this simulation all 200 societies make the transition within 60 periods. In any given period, societies with higher *B* have higher levels of $z_{t}$.

**Fig. 3:**
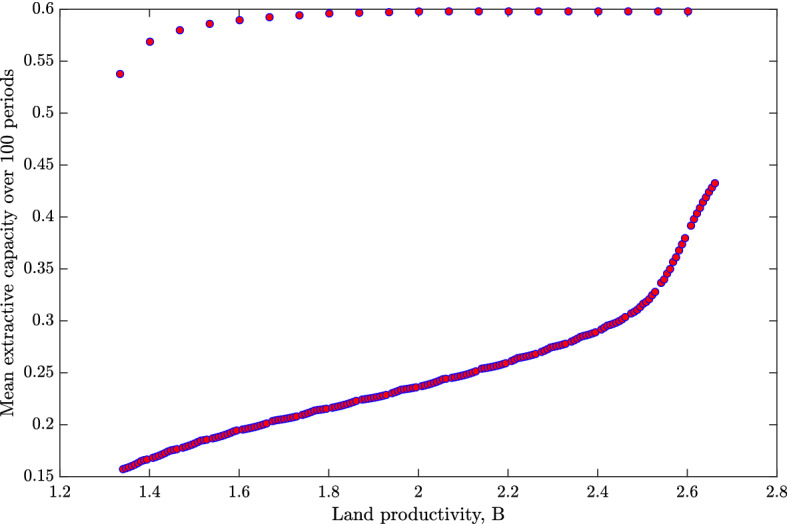
Plot showing the cross-sectional relationship between land productivity, *B*, and mean extractive capacity over 100 periods, based on the same simulation as in Fig. 2. Each circle represents one society

Figure 3 illustrates the cross-sectional relationship between land productivity and a cumulative statehood measure, namely mean extractive capacity over the 100 periods. The 20 societies with the highest levels of statehood are those that experienced a positive shock. By assumption, these have levels of *B* distributed across the same interval as the remaining 180, and thus show little association between land productivity and state history.^16^ Among the remainder, however, we see a clear positive relationship between land productivity and mean extractive capacity, such that the highest levels of statehood are found in societies with the highest land productivity.

### Cross-country evidence from Eurasia

Next we explore if this pattern is consistent with cross-country data. We focus on the continent of Eurasia, where most state building has spread from a couple of centers (see discussion below). We use accumulated State Antiquity over different periods from 3500 BCE to 1500 CE from Borcan et al. (2018) to measure statehood (corresponding to mean extractive capacity over time in the simulation). We use the Caloric Suitability Index (CSI) from Galor and Özak (2016) to measure land productivity. (See Sect. 2 of the Appendices for more details about the data.)

Table 1 presents results from regressing State Antiquity on CSI for different subsamples, namely countries which developed statehood before and after different temporal cutoffs. Columns (1)–(3) consider 450 CE, a common benchmark for the end of the classical-age state building era (see, e.g., Mayshar et al., 2020). Columns (4)–(9) consider 1000 BCE, an earlier point at which much fewer countries had begun to develop statehood.

**Table 1:** Agricultural productivity and statehood: countries with late and early state development

	Dependent variable is State Antiquity over the period								
	3500 BCE to 1500 CE		450 CE to 1500 CE	3500 BCE to 1500 CE		1000 BCE to 1500 CE	3500 BCE to 450 CE		1000 BCE to 450 CE
	(1)	(2)	(3)	(4)	(5)	(6)	(7)	(8)	(9)
Galor–Özak CSI	30.38**	− 90.00**	18.96	69.10***	− 23.24	18.14	28.59**	− 44.05	− 3.22
	(11.36)	(35.33)	(11.88)	(24.83)	(40.90)	(30.87)	(10.76)	(35.65)	(26.64)
State Antiquity 3500 BCE-450 CE			0.00 (0.05)						
State Antiquity 3500-1000 BCE						0.16 (0.14)			0.17 (0.11)
$\hbox {R}^2$	0.14	0.17	0.09	0.13	0.01	0.07	0.07	0.05	0.11
Number of obs.	23	52	52	56	19	19	56	19	19
Arrival of statehood	After 450 CE	Before 450 CE	Before 450 CE	After 1000 BCE	Before 1000 BCE	Before 1000 BCE	After 1000 BCE	Before 1000 BCE	Before 1000 BCE

Ordinary least squares regressions across Eurasian countries with robust standard errors in parentheses. The dependent variable is accumulated State Antiquity over different periods. The sample is split between countries which developed statehood early and late, respectively: before and after 450 CE in columns (1)–(3), and before and after 1000 BCE in columns (4)–(9)

*$$p <0.10$$; **$$p <0.05$$; ***$$p <0.01$$

Consider first columns (1), (4), and (7) in Table 1, which use samples of countries with relatively late state development. Here we find a positive and significant correlation between the Galor–Özak CSI index and statehood. The relationship among countries with earlier state development in the remaining columns is mostly insignificant, at least when controlling for existing state development up until the cutoff year; see columns (3), (6), and (9). This is consistent with the simulation results in Fig. 3. That is, the relationship between accumulated statehood and land productivity tends to be positive for countries that developed statehood later, and close to zero for those with early statehood.

**Fig. 4:**
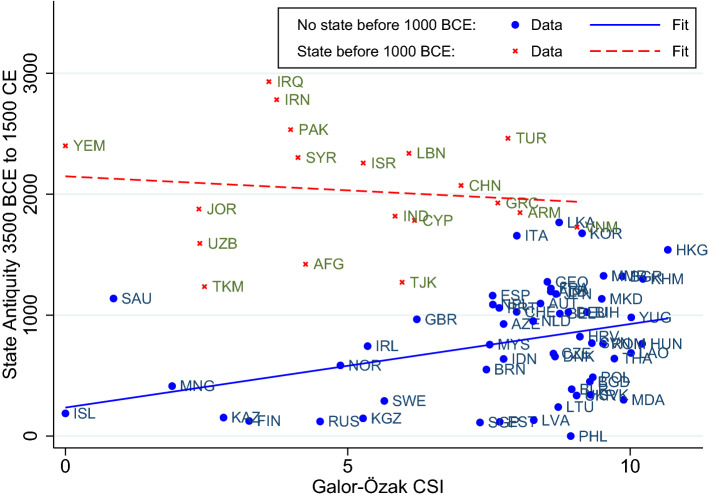
Plot showing the relationship between statehood and the Galor–Özak CSI index for Eurasian countries that already had some statehood before 1000 BCE, and those that did not

Figure 4 illustrates the relationship between land productivity and statehood for early and late state developers, using 1000 BCE as cutoff; cf. columns (4) and (5) in Table 1. Note that the pattern is qualitatively similar to the simulated one in Fig. 3.

Table 2 explores these cross-country data further when using 1000 BCE as cutoff for late and early state development, but using the full sample of Eurasian countries and instead interacting land productivity with an indicator for late state development. Column (1) first documents a negative but insignificant unconditional relationship between Galor–Özak CSI and statehood. This turns positive and significant in column (2), where we enter a Late Statehood Dummy, equal to one for countries which developed statehood after 1000 BCE. The Late Statehood Dummy itself carries a significant negative coefficient for obvious reasons.

**Table 2:** Agricultural productivity and statehood: interacting late statehood with agricultural productivity

	Dependent variable is State Antiquity 3500 BCE to 1500 CE					
	(1)	(2)	(3)	(4)	(5)	(6)
Galor–Özak CSI	− 58.76	46.41**	− 23.24	− 42.63	− 42.65	− 40.82
	(37.03)	(21.86)	(39.76)	(32.39)	(32.67)	(35.41)
Late Statehood Dummy		− 1383.62*** (132.09)	− 1912.32*** (338.10)	− 1766.34*** (307.02)	− 1752.91*** (280.72)	− 1736.35*** (296.55)
Late Statehood $\times$ Galor–Özak CSI			92.34* (47.00)	99.02*** (36.23)	98.07*** (34.65)	95.71** (38.25)
Distance from State Origin					− 0.01 (0.09)	− 0.02 (0.10)
Log Absolute Latitude						− 25.06 (169.25)
$\hbox {R}^2$	0.05	0.61	0.63	0.78	0.78	0.78
Number of obs.	75	75	75	75	75	75
Region fixed effects	No	No	No	Yes	Yes	Yes

Ordinary least squares regressions across Eurasian countries with robust standard errors in parentheses. The dependent variable is accumulated State Antiquity 3500 BCE to 1500 CE. Late Statehood is an indicator for a country not having a state before 1000 CE. Region Fixed Effects is a set of dummies for each of nine regions

*$$p <0.10$$; **$$p <0.05$$; ***$$p <0.01$$

In column (3) we interact the Late Statehood Dummy and the Galor–Özak CSI index. The interaction term comes out as positive and significant just below the 5% level. It stays positive and becomes much more precisely estimated in column (4), where we include region fixed effects. Column (5) also controls for the geodetic distance from country centroids to Baghdad or Beijing, whichever is closest, conjectured centers for state origins in Eurasia. Column (6) adds a control for Log Absolute Latitude. Throughout, the positive coefficient on the interaction term stays significant at the 5% level, or better. In other words, land productivity shows a positive association with statehood among countries that developed statehood later, just as we should expect.

As mentioned, we here focus on the Eurasian continent, since state building did not spread between Eurasia and other continents prior to 1500. When including the Americas, or the rest of the world, the results in Tables 1 and 2 tend to weaken. This seems consistent with the idea that land productivity should matter more when state building tools can be copied or imported more easily.

### Anecdotal evidence from Sweden

The data presented above end in 1500 CE, but state building continued after that, in particular in Northern Europe, which lagged behind the continent (cf. Fig. 4). Sweden offers some concrete examples of how rulers of younger states could use tax revenue to import state building after 1500.

As described by Ertman (1997, pp. 313–314), in 1538 Sweden’s first king Gustav I (or Gustav Vasa) hired a German minister, Conrad von Pyhy, to organize its central administration following a template from the Holy Roman Empire. From 1611, Gustavus Adolphus continued state centralization by borrowing from more recent German and Dutch models.

Architecture offers another example. The oldest and most famous castles and monuments from Sweden’s so-called Great Power era in the 17th century were designed by foreign architects, in particular Simon de la Vallée and Nicodemus Tessin the Elder, who acquired their skills on the continent (Stevens Curl & Wilson, 2015). There may be more important (and productive) aspects of state building than castles, but this does illustrate that skills related to state building could indeed be imported.

## Concluding remarks

There are many competing explanations of what caused the rise and spread of statehood, or social stratification more generally. The Surplus Theory posits that a non-producing elite could only be supported with a “surplus” supply of food. This surplus, goes the argument, arrived when land productivity rose in the wake of the Neolithic Revolution, i.e., when humans transitioned from food procurement through hunting and gathering to using agriculture. A different theory has been labelled the Appropriability Theory. It holds that the rise of states was rather about the arrival of new crops, which were easier for a ruling elite to confiscate.

This paper has presented a model which incorporates mechanisms related to those emphasized by both the Surplus and Appropriability Theories. A ruler extracts resources from a subject population, the size of which evolves over time in a Malthusian fashion, dependent on the ruler’s rate of extraction. The ruler can invest the extracted resources in what we call extractive and productive capacities. These complement each other in such a way that the model can give rise to multiple steady states holding constant land productivity and other exogenous factors. One steady state has low extractive capacity, a low extraction rate, and low population density and output; the other has high extractive capacity, a high extraction rate, and high population density and output.

Not only can the combination of extractive and productive capacities give rise multiple steady states. This paper has shown that both of these elements are needed for such multiplicity to arise. In that sense, the Surplus and Appropriability Theories, as modelled here, can generate richer theoretical results together than each theory on its own.

To illustrate the empirical relevance of the model we exploit its complementarity between land productivity and the return to state building. Intuitively, countries which develop statehood later are able to draw on the state knowledge accumulated by earlier states, and thus face a higher return to efforts and resources directed towards state building compared to countries which developed statehood from scratch. Therefore, among countries which transition into statehood relatively late, we should expect too see a positive association between land productivity and state antiquity, but not necessarily among earlier states. Evidence from across Eurasian countries supports this prediction.

### Supplementary Information

Below is the link to the electronic supplementary material.

Supplementary material 1 (pdf 188 KB)

## The ruler’s maximization problem

### Finding optimal $A_{t+1}$, $z_{t+1}$ and $\tau _{t}$

First note from (1) and (5) that output in period $t+1$ can be written

26 $$\begin{aligned} Y_{t+1}=(BA_{t+1})^{\alpha }\left[ \gamma (1-\tau _{t})Y_{t}\right] ^{1-\alpha }. \end{aligned}$$

Substituting $z_{t+1}={\underline{z}}+\phi x_{t}$, (7), and (26) into (8), we can write $U_{t}^{R}$ as a function of $A_{t+1}$, $x_{t}$, and $\tau _{t}$, namely

27 $$\begin{aligned} \begin{array}{ll} U_{t}^{R}&{}= (1-\beta )\ln \left( \tau _{t}z_{t}Y_{t}-\eta A_{t+1}^{\sigma }-x_{t}\right) \\ &{}\quad +\beta \ln ({\underline{z}}+\phi x_{t}) \\ &{}\quad +\alpha \beta \ln \left( A_{t+1}\right) \\ &{}\quad +\beta (1-\alpha )\ln (1-\tau _{t}) \\ &{}\quad +\Omega _{t} \end{array} \end{aligned}$$

where

$$\begin{aligned} \Omega _{t}=\alpha \beta \ln B+\beta (1-\alpha )\ln (Y_{t})+\beta (1-\alpha )\ln \gamma \end{aligned}$$

contains only variables taken as given by the ruler. The problem is to maximize (27) subject to $A_{t+1}\ge 0$, $\tau _{t}\ge 0$, $\tau _{t}\le 1$, $x_{t}\ge 0$, and $x_{t}\le ({\overline{z}}-{\underline{z}})/\phi$; the last two constraints correspond to $z_{t+1}\ge {\underline{z}}$ and $z_{t+1}\le {\overline{z}}$, respectively.

The first-order conditions for an interior solution state that $A_{t+1}$ and $\tau _{t}$ satisfy

28 $$\begin{aligned} (1-\beta )\left[ c_{t}^{R}\right] ^{-1}\eta \sigma A_{t+1}^{\sigma -1}=\alpha \beta \left[ A_{t+1}\right] ^{-1}, \end{aligned}$$

and

29 $$\begin{aligned} (1-\beta )\left[ c_{t}^{R}\right] ^{-1}z_{t}Y_{t}=\beta (1-\alpha )\left[ 1-\tau _{t}\right] ^{-1}, \end{aligned}$$

where $$c_{t}^{R}=\tau _{t}z_{t}Y_{t}-\eta A_{t+1}^{\sigma }-x_{t}$$; recall (7).

It is straightforward to see that the constraints $A_{t+1}\ge 0$, $\tau _{t}\ge 0$, and $\tau _{t}\le 1$ never bind, so (28) and (29) always give optimal $A_{t+1}$ and $\tau _{t}$ for any $x_{t}\in [0,( {\overline{z}}-{\underline{z}})/\phi ]$. Using (7), (28), and (29) we can solve for $\eta A_{t+1}^{\sigma }$ and $1-\tau _{t}$ as follows:

30 $$\begin{aligned} \eta A_{t+1}^{\sigma }=\left[ \frac{\alpha \beta }{\sigma (1-\alpha \beta )+\alpha \beta }\right] \left( z_{t}Y_{t}-x_{t}\right) , \end{aligned}$$

31 $$\begin{aligned} 1-\tau _{t}=\left[ \frac{\beta \sigma (1-\alpha )}{\sigma (1-\alpha \beta )+\alpha \beta }\right] \left( 1-\frac{x_{t}}{z_{t}Y_{t}}\right) . \end{aligned}$$

Also, using (7), (30), and (31) we can write the ruler’s consumption as

32 $$\begin{aligned} c_{t}^{R}=\left[ \frac{\sigma (1-\beta )}{\sigma (1-\alpha \beta )+\alpha \beta }\right] \left( z_{t}Y_{t}-x_{t}\right) . \end{aligned}$$

Below we use (30) to (32) to find the optimal choices of $A_{t+1}$ and $\tau _{t}$ for three cases: when $x_{t}=0$; when $x_{t}=( {\overline{z}}-{\underline{z}})/\phi$; and when $0<x_{t}<({\overline{z}}- {\underline{z}})/\phi$.

#### Corner solutions where $x_{t}=0$

If the marginal effect on $U_{t}^{R}$ from an increase in $x_{t}$ is negative when $x_{t}=0$, then $x_{t}=0$ is optimal. This happens when

33 $$\begin{aligned} \left. \frac{\partial U_{t}^{R}}{\partial x_{t}}\right| _{x_{t}=0}=-(1-\beta )\left[ \tau _{t}z_{t}Y_{t}-\eta A_{t+1}^{\sigma } \right] ^{-1}+\phi \beta \left[ {\underline{z}}\right] ^{-1}<0. \end{aligned}$$

Using (30) and (31) we see that $\tau _{t}z_{t}Y_{t}-\eta A_{t+1}^{\sigma }$ is simply the expression for $c_{t}^{R}$ in (32), evaluated at $x_{t}=0$. Thus, the inequality in (33) can be written

34 $$\begin{aligned} (1-\beta )\left( \left[ \frac{\sigma (1-\beta )}{\sigma (1-\alpha \beta )+\alpha \beta }\right] z_{t}Y_{t}\right) ^{-1}>\phi \beta {\underline{z}}^{-1} , \end{aligned}$$

which translates to $z_{t}Y_{t}<{\underline{X}}$, where ${\underline{X}}$ is given by (13).

It thus follows that if $z_{t}Y_{t}<{\underline{X}}$, then $x_{t}=0$. Moreover, optimal $A_{t+1}$ and $\tau _{t}$ can be found by setting $x_{t}=0$ in (30) and (31). This gives the bottom rows of (14) and (43) below.

#### Corner solutions where $x_{t}=({\overline{z}}-{\underline{z}} )/\phi$

If the marginal effect on $U_{t}^{R}$ from an increase in $x_{t}$ is positive when $x_{t}=({\overline{z}}-{\underline{z}})/\phi$, then $x_{t}=( {\overline{z}}-{\underline{z}})/\phi$ is optimal. This happens when

35 $$\begin{aligned} \left. \frac{\partial U_{t}^{R}}{\partial x_{t}}\right| _{x_{t}=\frac{ {\overline{z}}-{\underline{z}}}{\phi }}=-(1-\beta )\left[ \tau _{t}z_{t}Y_{t}-\eta A_{t+1}^{\sigma }-\left( \frac{{\overline{z}}-{\underline{z}} }{\phi }\right) \right] ^{-1}+\phi \beta {\overline{z}}^{-1}>0. \end{aligned}$$

The expression in square brackets in (35) equals $c_{t}^{R}$ in (32), evaluated at $x_{t}=({\overline{z}}-{\underline{z}})/\phi$. Substituted into (35), this gives

36 $$\begin{aligned} (1-\beta )\left[ \left( \frac{\sigma (1-\beta )}{\sigma (1-\alpha \beta )+\alpha \beta }\right) \left( z_{t}Y_{t}-\frac{{\overline{z}}-{\underline{z}}}{ \phi }\right) \right] ^{-1}<\phi \beta {\overline{z}}^{-1}, \end{aligned}$$

or

37 $$\begin{aligned} {\overline{z}}<\left( \frac{\sigma \beta }{\sigma (1-\alpha \beta )+\alpha \beta }\right) \left[ \phi z_{t}Y_{t}-\left( {\overline{z}}-{\underline{z}} \right) \right] , \end{aligned}$$

which can in turn be simplified to $z_{t}Y_{t}>{\overline{X}}$, where ${\overline{X}}$ is given by (12).

To sum up, if $z_{t}Y_{t}>{\overline{X}}$, then $x_{t}=({\overline{z}}- {\underline{z}})/\phi$ and optimal $A_{t+1}$ and $\tau _{t}$ can be found by setting $x_{t}=({\overline{z}}-{\underline{z}})/\phi$ in (30) and (31). This gives the top rows of (14) and (43) below.

#### Interior solutions

Consider next interior solutions for $x_{t}$, which can be found when $z_{t}Y_{t}\in ({\underline{X}},{\overline{X}})$, and are derived from the first-order condition

38 $$\begin{aligned} (1-\beta )\left[ c_{t}^{R}\right] ^{-1}=\phi \beta \left[ {\underline{z}}+\phi x_{t}\right] ^{-1}\,, \end{aligned}$$

where $c_{t}^{R}$ is given by (32). We can use (32) and (38) to find optimal $x_{t}$, but we are rather interested in the associated expression for $z_{t+1}$. Since $z_{t+1}={\underline{z}}+\phi x_{t}$ in an interior solution for $x_{t}$, we can write the first-order condition in (38) as

39 $$\begin{aligned} (1-\beta )\left( \left[ \frac{\sigma (1-\beta )}{\sigma (1-\alpha \beta )+\alpha \beta }\right] \left[ z_{t}Y_{t}-\left( \frac{z_{t+1}-{\underline{z}} }{\phi }\right) \right] \right) ^{-1}=\phi \beta \left[ z_{t+1}\right] ^{-1} . \end{aligned}$$

This can be solved for $z_{t+1}$ to give

40 $$\begin{aligned} z_{t+1}={\underline{z}}+\phi x_{t}=\left( \frac{\beta \sigma }{\beta \sigma (1-\alpha )+\sigma +\alpha \beta }\right) \left[ \phi z_{t}Y_{t}+{\underline{z}}\right] , \end{aligned}$$

which is the middle row of (15).

Using (40), we can also derive the associated solutions for $\tau _{t}$ and $A_{t+1}$. Dividing (28) by (38), and rearranging, investment in next period’s technology becomes

41 $$\begin{aligned} \eta A_{t+1}^{\sigma }=\frac{\alpha }{\sigma \phi }\left( {\underline{z}}+\phi x_{t}\right) =\left( \frac{\alpha \beta }{\beta \sigma (1-\alpha )+\sigma +\alpha \beta }\right) \left( z_{t}Y_{t}+\frac{{\underline{z}}}{\phi }\right) , \end{aligned}$$

where the second equality follows from (40). Solving (41) for $A_{t+1}$ gives the middle row in (43) below.

Similarly, dividing (29) by (38), using (40), and rearranging, gives

42 $$\begin{aligned} (1-\tau _{t})z_{t}Y_{t}=\left( \frac{1-\alpha }{\phi }\right) \left[ {\underline{z}}+\phi x_{t}\right] =\left( \frac{\beta \sigma (1-\alpha )}{ \beta \sigma (1-\alpha )+\sigma +\alpha \beta }\right) \left( z_{t}Y_{t}+ \frac{{\underline{z}}}{\phi }\right) \end{aligned}$$

which can be solved to give the middle row in (14).

### Complete characterization of the solution

To sum up, we can write the optimal expressions for $\tau _{t}$ as in (14), for $z_{t+1}$ as in (15), and the optimal expression for $A_{t+1}$ can be written

43 $$\begin{aligned} A_{t+1}=\left\{ \begin{array}{lll} \left[ \frac{1}{\eta }\left( \frac{\alpha \beta }{\sigma (1-\alpha \beta )+\alpha \beta }\right) \left[ z_{t}Y_{t}-\left( \frac{{\overline{z}}- {\underline{z}}}{\phi }\right) \right] \right] ^{\frac{1}{\sigma }} &{} \text {if} &{} z_{t}Y_{t}\ge {\overline{X}}, \\ \left[ \frac{1}{\eta }\left( \frac{\alpha \beta }{\beta \sigma (1-\alpha )+\sigma +\alpha \beta }\right) \left( z_{t}Y_{t}+\frac{{\underline{z}}}{\phi } \right) \right] ^{\frac{1}{\sigma }} &{} \text {if} &{} z_{t}Y_{t}\in \left[ {\underline{X}},{\overline{X}}\right] , \\ \left[ \frac{1}{\eta }\left( \frac{\alpha \beta }{\sigma (1-\alpha \beta )+\alpha \beta }\right) z_{t}Y_{t}\right] ^{\frac{1}{\sigma }} &{} \text {if } &{} z_{t}Y_{t}\le {\underline{X}}. \end{array} \right. \end{aligned}$$

## Dynamics of $Y_{t}$

This section finds an expression for $Y_{t+1}$ in terms of $Y_{t}$ and $z_{t}$ (and exogenous variables, such as *B*). Consider first the case when $z_{t}Y_{t}\in \left[ {\underline{X}},{\overline{X}}\right]$, meaning neither of the constraints on $z_{t+1}$ binds. Using (14), it is then seen that

44 $$\begin{aligned} \gamma (1-\tau _{t})Y_{t}=\frac{\gamma }{\phi z_{t}}\left[ \frac{\beta \sigma (1-\alpha )}{\beta \sigma (1-\alpha )+\sigma +\alpha \beta }\right] \left( \phi z_{t}Y_{t}+{\underline{z}}\right) . \end{aligned}$$

Now (26), (43), and (44) tell us that

45 $$\begin{aligned} \begin{array}{c} Y_{t+1}=B^{\alpha }\underset{A_{t+1}^{\alpha }}{\underbrace{\left( \frac{1}{ \eta \phi }\right) ^{\frac{\alpha }{\sigma }}\left( \frac{\alpha \beta }{ \beta \sigma (1-\alpha )+\sigma +\alpha \beta }\right) ^{\frac{\alpha }{ \sigma }}\left( \phi z_{t}Y_{t}+{\underline{z}}\right) ^{\frac{\alpha }{\sigma }}}} \\ \qquad \underset{\left[ \gamma (1-\tau _{t})Y_{t}\right] ^{1-\alpha }}{\times \underbrace{\left( \frac{\gamma }{\phi z_{t}}\right) ^{1-\alpha }\left( \frac{\beta \sigma (1-\alpha )}{\beta \sigma (1-\alpha )+\sigma +\alpha \beta }\right) ^{1-\alpha }\left[ \phi z_{t}Y_{t}+{\underline{z}}\right] ^{1-\alpha }}}. \end{array} \end{aligned}$$

To simplify this expression, first define

46 $$\begin{aligned} \rho =\frac{\alpha }{\sigma }+1-\alpha \in (0,1) \end{aligned}$$

and

47 $$\begin{aligned} D=\left[ \frac{\alpha }{\eta \sigma (1-\alpha )}\right] ^{\frac{\alpha }{ \sigma }}\gamma ^{1-\alpha }\left( \frac{1}{\phi }\right) ^{\rho }\left[ \frac{\beta \sigma (1-\alpha )}{\beta \sigma (1-\alpha )+\sigma +\alpha \beta }\right] ^{\rho }, \end{aligned}$$

where we note that $\rho <1$ follows from $\sigma >1$. Using (46) and (47), we can rewrite (45) more compactly as

48 $$\begin{aligned} Y_{t+1}=DB^{\alpha }z_{t}^{\alpha -1}\left( \phi z_{t}Y_{t}+{\underline{z}} \right) ^{\rho }, \end{aligned}$$

which is the middle row of (16).

Consider next the case when $z_{t}Y_{t}>{\overline{X}}$. From (14), it now follows that

49 $$\begin{aligned} \gamma (1-\tau _{t})Y_{t}=\frac{\gamma }{\phi z_{t}}\left[ \frac{\beta \sigma (1-\alpha )}{\sigma (1-\alpha \beta )+\alpha \beta }\right] \left( \phi z_{t}Y_{t}+{\underline{z}}-{\overline{z}}\right) . \end{aligned}$$

Following similar steps as we followed above for the interior solution, we can use (26), (43), and (49) to show that

50 $$\begin{aligned} Y_{t+1}={\widehat{D}}B^{\alpha }z_{t}^{\alpha -1}\left( \phi z_{t}Y_{t}+ {\underline{z}}-{\overline{z}}\right) ^{\rho }, \end{aligned}$$

where

51 $$\begin{aligned} {\widehat{D}}=\left[ \frac{\alpha }{\eta \sigma (1-\alpha )}\right] ^{\frac{ \alpha }{\sigma }}\gamma ^{1-\alpha }\left( \frac{1}{\phi }\right) ^{\rho } \left[ \frac{\beta \sigma (1-\alpha )}{\sigma (1-\alpha \beta )+\alpha \beta }\right] ^{\rho }, \end{aligned}$$

and (recall) $\rho$ is given by (46).

Finally, for the case when $z_{t}Y_{t}<{\underline{X}}$, we can use (14) again to see that

52 $$\begin{aligned} \gamma (1-\tau _{t})Y_{t}=\gamma \left[ \frac{\beta \sigma (1-\alpha )}{ \sigma (1-\alpha \beta )+\alpha \beta }\right] Y_{t}. \end{aligned}$$

Applying (26), (43), and (52) some algebra shows that

53 $$\begin{aligned} Y_{t+1}={\widehat{D}}B^{\alpha }z_{t}^{\frac{\alpha }{\sigma }}\left( \phi Y_{t}\right) ^{\rho }={\widehat{D}}B^{\alpha }z_{t}^{\alpha -1}\left( \phi z_{t}Y_{t}\right) ^{\rho }, \end{aligned}$$

where $\rho$ and ${\widehat{D}}$ are given by (46) and (51).

Finally, using (46), (47), and (51) we can define $\kappa$ as

54 $$\begin{aligned} \kappa =\frac{{\widehat{D}}}{D}=\left[ \frac{\beta \sigma (1-\alpha )+\sigma +\alpha \beta }{\sigma (1-\alpha \beta )+\alpha \beta }\right] ^{\rho }>1 . \end{aligned}$$

Now, using (50), (53), and substituting for ${\widehat{D}} =\kappa D$, we arrive at the top and bottom rows of (16).

## The phase diagram

### The ($z_{t+1}=z_{t}$)-locus

The following can be seen directly from (15): for $z_{t}Y_{t}\le {\underline{X}}$, it holds that $z_{t+1}=z_{t}$ when $z_{t}={\underline{z}}$; for $z_{t}Y_{t}\ge {\overline{X}}$, it holds that $z_{t+1}=z_{t}$ when $z_{t}= {\overline{z}}$; for $z_{t}Y_{t}\in \left[ {\underline{X}},{\overline{X}}\right]$ , it holds that $z_{t+1}=z_{t}$ when $z_{t}=\left( \frac{\beta \sigma }{ \beta \sigma (1-\alpha )+\sigma +\alpha \beta }\right) \left[ \phi z_{t}Y_{t}+{\underline{z}}\right]$, or $z_{t}=\beta \sigma {\underline{z}} /\{\beta \sigma (1-\alpha )+\sigma +\alpha \beta -\beta \sigma \phi Y_{t}\}$ . In sum, the ($z_{t+1}=z_{t}$)-locus can be written

55 $$\begin{aligned} z_{t}=\left\{ \begin{array}{lll} {\overline{z}} &{} \text {if} &{} z_{t}Y_{t}\ge {\overline{X}}, \\ \frac{\beta \sigma {\underline{z}}}{\beta \sigma (1-\alpha )+\sigma +\alpha \beta -\beta \sigma \phi Y_{t}} &{} \text {if} &{} z_{t}Y_{t}\in \left[ {\underline{X}},{\overline{X}}\right] , \\ {\underline{z}} &{} \text {if } &{} z_{t}Y_{t}\le {\underline{X}}. \end{array} \right. \end{aligned}$$

The (inverse of) (55) is graphed in Fig. 1 as a three-segment solid blue curve.

### The ($Y_{t+1}=Y_{t}$)-locus

From (16) we learn the following: for $z_{t}Y_{t}\le {\underline{X}}$, it holds that $Y_{t+1}=Y_{t}$ when $Y_{t}=\left[ \kappa DB^{\alpha }\phi ^{\rho }z_{t}^{\alpha /\sigma }\right] ^{1/(1-\rho )}$ (from using $\rho =\alpha /\sigma +1-\alpha$); for $z_{t}Y_{t}\ge {\overline{X}}$, it holds that $Y_{t+1}=Y_{t}$ when $Y_{t}=\xi (z_{t},B)$, defined from $\xi (z_{t},B)=\kappa DB^{\alpha }z_{t}^{\alpha -1}\left[ \phi z_{t}\xi (z_{t},B)+ {\underline{z}}-{\overline{z}}\right] ^{\rho }$; for $z_{t}Y_{t}\in \left[ {\underline{X}},{\overline{X}}\right]$, it holds that $Y_{t+1}=Y_{t}$ when $Y_{t}=\vartheta (z_{t},B)$, defined from $\vartheta (z_{t},B)=\kappa DB^{\alpha }z_{t}^{\alpha -1}\left[ \phi z_{t}\vartheta (z_{t},B)+{\underline{z}}\right] ^{\rho }$. To summarize, the ($Y_{t+1}=Y_{t}$)-locus can be written

56 $$\begin{aligned} Y_{t}=\left\{ \begin{array}{lll} \xi (z_{t},B) &{} \text {if} &{} z_{t}Y_{t}\ge {\overline{X}}, \\ \vartheta (z_{t},B) &{} \text {if} &{} z_{t}Y_{t}\in \left[ {\underline{X}}, {\overline{X}}\right] , \\ \left[ \kappa DB^{\alpha }\phi ^{\rho }z_{t}^{\frac{\alpha }{\sigma }}\right] ^{\frac{1}{1-\rho }} &{} \text {if } &{} z_{t}Y_{t}\le {\underline{X}}. \end{array} \right. \end{aligned}$$

The red solid curves in Fig. 1 show the graphs of the three different segments of the ($Y_{t+1}=Y_{t}$)-locus in (56).

### Change in configuration when changing *B*

Note that the ($z_{t+1}=z_{t}$)-locus does not depend on *B*. It is easy to see, from the definitions above, that $\xi (z_{t},B)$ and $\vartheta (z_{t},B)$ are strictly increasing in *B*, that $\lim _{B\rightarrow \infty }\xi (z_{t},B)=\lim _{B\rightarrow \infty }\vartheta (z_{t},B)=\infty$, and that $\xi (z_{t},0)=\vartheta (z_{t},0)=0$. It follows that we can adjust *B* to shift the ($Y_{t+1}=Y_{t}$)-locus to alter the configuration of the two-dimensional dynamical system. When *B* is sufficiently small the ($Y_{t+1}=Y_{t}$)- and ($z_{t+1}=z_{t}$)-loci intersect only once, and this unique intersection lies in the region where $z_{t}Y_{t}<{\underline{X}}$. When *B* is sufficiently large the two loci also intersect only once, now in the region where $z_{t}Y_{t}>{\overline{X}}$.

## Closing down channels

### Closing down investment in extractive capacity

In this setting, the first-order conditions for $A_{t+1}$ and $\tau _{t}$ can be written

57 $$\begin{aligned} (1-\beta )\left[ \tau _{t}{\widetilde{z}}Y_{t}-\eta A_{t+1}^{\sigma }\right] ^{-1}\eta \sigma A_{t+1}^{\sigma -1}=\alpha \beta \left[ A_{t+1}\right] ^{-1} , \end{aligned}$$

and

58 $$\begin{aligned} (1-\beta )\left[ \tau _{t}{\widetilde{z}}Y_{t}-\eta A_{t+1}^{\sigma }\right] ^{-1}{\widetilde{z}}Y_{t}=\beta (1-\alpha )\left[ 1-\tau _{t}\right] ^{-1} . \end{aligned}$$

Solving for $\tau _{t}$ gives the same expression as in the bottom row in ( 14). The expression for $\eta A_{t+1}^{\sigma }$ becomes identical to that in (30), but with $x_{t}=0$ and $z_{t}={\widetilde{z}}$, i.e.,

59 $$\begin{aligned} \eta A_{t+1}^{\sigma }=\left[ \frac{\alpha \beta }{\sigma (1-\alpha \beta )+\alpha \beta }\right] {\widetilde{z}}Y_{t}. \end{aligned}$$

Using $Y_{t+1}=(BA_{t+1})^{\alpha }L_{t+1}^{1-\alpha }$ and $L_{t+1}=\gamma (1-\tau _{t})Y_{t}$, together with the expressions for $\tau _{t}$ in the bottom row in (14), and $A_{t+1}$ in (59), some algebra shows that $Y_{t+1}=GY_{t}^{\rho }$, where

60 $$\begin{aligned} G=B^{\alpha }\left[ \frac{\alpha {\widetilde{z}}}{\eta \sigma (1-\alpha )} \right] ^{\frac{\alpha }{\sigma }}\gamma ^{1-\alpha }\left[ \frac{\beta \sigma (1-\alpha )}{\sigma (1-\alpha \beta )+\alpha \beta }\right] ^{\rho } \end{aligned}$$

and (recall) $$\rho =(\alpha /\sigma )+1-\alpha <1$$.

Using (51), it can also be seen that $G=B^{\alpha }{\widehat{D}}\phi ^{\rho }{\widetilde{z}}^{\frac{\alpha }{\sigma }}$, which shows that $Y_{t+1}=GY_{t}^{\rho }$ can be derived from (16), setting $z_{t}= {\widetilde{z}}$. That is, the dynamics in the model without investment in extractive capacity coincide with those in the benchmark model in the relevant corner solution.

### Closing down investment in productive capacity

In the model without investment in productive capacity, the first-order condition for $\tau _{t}$ (which always holds with equality) becomes

61 $$\begin{aligned} (1-\beta )\left[ \tau _{t}z_{t}Y_{t}-x_{t}\right] ^{-1}z_{t}Y_{t}=\beta (1-\alpha )\left[ 1-\tau _{t}\right] ^{-1}. \end{aligned}$$

where we have used $c_{t}^{R}=\tau _{t}z_{t}Y_{t}-x_{t}$. It can be seen from (61) that $\tau _{t}$ can be written

62 $$\begin{aligned} \tau _{t}=1-\frac{\beta (1-\alpha )}{1-\alpha \beta }\left( 1-\frac{x_{t}}{ z_{t}Y_{t}}\right) . \end{aligned}$$

#### Dynamics for extractive capacity, $z_{t}$

The optimal choice of $x_{t}$ (which determines $z_{t+1}$) involves corner solutions. If the marginal effect on $U_{t}^{R}$ from an increase in $x_{t}$ is negative when $x_{t}=0$, then $x_{t}=0$ is optimal. The condition for this can be written:

63 $$\begin{aligned} \left. \frac{\partial U_{t}^{R}}{\partial x_{t}}\right| _{x_{t}=0}=-(1-\beta )\left[ \tau _{t}z_{t}Y_{t}\right] ^{-1}+\phi \beta \left[ {\underline{z}}\right] ^{-1}<0. \end{aligned}$$

If $x_{t}=0$, we see from (62) that

64 $$\begin{aligned} \tau _{t}=\frac{1-\beta }{1-\alpha \beta }. \end{aligned}$$

Using (64) and (63), we see that $x_{t}=0$ is the ruler’s optimal choice when $z_{t}Y_{t}<{\underline{X}}$, where ${\underline{X}}$ is given by (25). That is, when $z_{t}Y_{t}<{\underline{X}}$, it holds that $x_{t}=0$ and $z_{t+1}={\underline{z}}$.

Next, if the marginal effect on $U_{t}^{R}$ from an increase in $x_{t}$ is positive when $z_{t+1}={\overline{z}}$, then $x_{t}=({\overline{z}}-{\underline{z}} )/\phi$ is optimal. The condition for this can be written:

65 $$\begin{aligned} \left. \frac{\partial U_{t}^{R}}{\partial x_{t}}\right| _{x_{t}=\frac{ {\overline{z}}-{\underline{z}}}{\phi }}=-(1-\beta )\left[ \tau _{t}z_{t}Y_{t}-\left( \frac{{\overline{z}}-{\underline{z}}}{\phi }\right) \right] ^{-1}+\phi \beta \left[ {\overline{z}}\right] ^{-1}>0. \end{aligned}$$

Evaluating (62) at $x_{t}=({\overline{z}}-{\underline{z}})/\phi$ gives

66 $$\begin{aligned} \tau _{t}=1-\frac{\beta (1-\alpha )}{1-\alpha \beta }\left[ 1-\frac{1}{ z_{t}Y_{t}}\left( \frac{{\overline{z}}-{\underline{z}}}{\phi }\right) \right] . \end{aligned}$$

Now (65) and (66) show that $x_{t}=({\overline{z}}- {\underline{z}})/\phi$ is optimal when $z_{t}Y_{t}>{\overline{X}}$, where ${\overline{X}}$ is given by (24). That is, when $z_{t}Y_{t}>{\overline{X}}$, it holds that $x_{t}=({\overline{z}}-{\underline{z}})/\phi$ and $z_{t+1}= {\overline{z}}$.

An interior solutions for $x_{t}$, which can be found when $z_{t}Y_{t}\in ( {\underline{X}},{\overline{X}})$, can be derived from the first-order condition

67 $$\begin{aligned} (1-\beta )\left[ \tau _{t}z_{t}Y_{t}-x_{t}\right] ^{-1}=\phi \beta \left[ {\underline{z}}+\phi x_{t}\right] ^{-1}. \end{aligned}$$

From (62) and (67) we find an expression for $z_{t+1}= {\underline{z}}+\phi x_{t}$ when $z_{t}Y_{t}\in ({\underline{X}},{\overline{X}})$, namely

68 $$\begin{aligned} z_{t+1}=\frac{\beta \left( \phi z_{t}Y_{t}+{\underline{z}}\right) }{1+\beta (1-\alpha )}. \end{aligned}$$

Thus, we can write

69 $$\begin{aligned} z_{t+1}=\left\{ \begin{array}{lll} {\overline{z}} &{} \text {if} &{} z_{t}Y_{t}\ge {\overline{X}}, \\ \frac{\beta \left( \phi z_{t}Y_{t}+{\underline{z}}\right) }{1+\beta (1-\alpha ) } &{} \text {if} &{} z_{t}Y_{t}\in \left[ {\underline{X}},{\overline{X}}\right] \text { ,} \\ {\underline{z}} &{} \text {if } &{} z_{t}Y_{t}\le {\underline{X}}, \end{array} \right. \end{aligned}$$

where we recall that the respective corner solutions coincide with the interior solution when $z_{t}Y_{t}={\underline{X}}$ and $z_{t}Y_{t}={\overline{X}}$.

#### Dynamics for output, $Y_{t}$

To find the dynamics for output, we use $Y_{t+1}=B^{\alpha }L_{t+1}^{1-\alpha }$ and $L_{t+1}=\gamma (1-\tau _{t})Y_{t}$; see (23 ). When $z_{t}Y_{t}<{\overline{X}}$, we can use the expression for $\tau _{t}$ in (64) to write

70 $$\begin{aligned} L_{t+1}=\gamma (1-\tau _{t})Y_{t}=\gamma \left[ \frac{\beta \left( 1-\alpha \right) }{1-\alpha \beta }\right] Y_{t}, \end{aligned}$$

which gives

71 $$\begin{aligned} Y_{t+1}=B^{\alpha }\left[ \frac{\gamma \beta \left( 1-\alpha \right) }{ 1-\alpha \beta }\right] ^{1-\alpha }Y_{t}^{1-\alpha }. \end{aligned}$$

When $z_{t}Y_{t}>{\overline{X}}$, we see from (66) that

72 $$\begin{aligned} L_{t+1}=\gamma (1-\tau _{t})Y_{t}=\frac{\gamma \beta (1-\alpha )}{1-\alpha \beta }\left[ Y_{t}-\frac{1}{z_{t}}\left( \frac{{\overline{z}}-{\underline{z}}}{ \phi }\right) \right] , \end{aligned}$$

which gives

73 $$\begin{aligned} Y_{t+1}=B^{\alpha }\left[ \frac{\gamma \beta (1-\alpha )}{1-\alpha \beta } \right] ^{1-\alpha }\left[ Y_{t}-\frac{1}{z_{t}}\left( \frac{{\overline{z}}- {\underline{z}}}{\phi }\right) \right] ^{1-\alpha }. \end{aligned}$$

When $z_{t}Y_{t}\in ({\underline{X}},{\overline{X}})$, we use the expression for $z_{t+1}$ in (68), which applies when $z_{t}Y_{t}\in ({\underline{X}}, {\overline{X}})$. Together with $z_{t+1}={\underline{z}}+\phi x_{t}$ this gives an expression for $x_{t}$, which can be substituted into (62) to show that

74 $$\begin{aligned} L_{t+1}=\gamma (1-\tau _{t})Y_{t}=\frac{\gamma \beta \left( 1-\alpha \right) }{1+\beta \left( 1-\alpha \right) }\left( \frac{\phi z_{t}Y_{t}+{\underline{z}} }{\phi z_{t}}\right) . \end{aligned}$$

Using $Y_{t+1}=B^{\alpha }L_{t+1}^{1-\alpha }$ and (74) gives

75 $$\begin{aligned} Y_{t+1}=B^{\alpha }\left[ \frac{\gamma \beta \left( 1-\alpha \right) }{ 1+\beta \left( 1-\alpha \right) }\right] ^{1-\alpha }\left( \frac{\phi z_{t}Y_{t}+{\underline{z}}}{\phi z_{t}}\right) ^{1-\alpha }. \end{aligned}$$

In sum, (71), (73), and (75) can be written in the same way as in (16), but with $\rho$ replaced by $1-\alpha$:

76 $$\begin{aligned} Y_{t+1}=\Psi (Y_{t},z_{t},B)\equiv \left\{ \begin{array}{lll} \kappa DB^{\alpha }z_{t}^{\alpha -1}\left[ \phi z_{t}Y_{t}+{\underline{z}}- {\overline{z}}\right] ^{1-\alpha } &{} \text {if} &{} z_{t}Y_{t}\ge {\overline{X}} , \\ DB^{\alpha }z_{t}^{\alpha -1}\left[ \phi z_{t}Y_{t}+{\underline{z}}\right] ^{1-\alpha } &{} \text {if} &{} z_{t}Y_{t}\in \left[ {\underline{X}},{\overline{X}} \right] , \\ \kappa DB^{\alpha }\left( \phi Y_{t}\right) ^{1-\alpha } &{} \text {if } &{} z_{t}Y_{t}\le {\underline{X}}, \end{array} \right. \end{aligned}$$

and with the following new definitions of *D* and $\kappa$:

77 $$\begin{aligned} \begin{array}{cc} D&{}= \left( \frac{\gamma \beta \left( 1-\alpha \right) }{\phi \left[ 1+\beta \left( 1-\alpha \right) \right] }\right) ^{1-\alpha }, \\ \kappa &{}= \left[ \frac{1+\beta \left( 1-\alpha \right) }{1-\alpha \beta } \right] ^{1-\alpha }. \end{array} \end{aligned}$$

## Proof of propositions

### Proof of Proposition 1

(a) Let ${\widehat{B}}$ be defined as the level of *B* that generates ${\underline{z}}{\underline{Y}}={\underline{X}}$. From (17) follows that

78 $$\begin{aligned} {\widehat{B}}=\frac{1}{{\underline{z}}}\left[ \frac{{\underline{X}}^{1-\rho }}{ \kappa D\phi ^{\rho }}\right] ^{\frac{1}{\alpha }}=\frac{1}{{\underline{z}}} \left[ \frac{{\underline{X}}}{\kappa D\left( \phi {\underline{X}}\right) ^{\rho } }\right] ^{\frac{1}{\alpha }}>0, \end{aligned}$$

where ${\underline{X}}$ is given by (13). Note from (17) that ${\underline{Y}}$ is increasing in *B*. By implication, $B<{\widehat{B}}$ is equivalent to ${\underline{z}}{\underline{Y}}<{\underline{X}}$.

(b) Let $\widehat{{\widehat{B}}}$ be defined as the level of *B* that generates ${\overline{z}}{\overline{Y}}={\overline{X}}$. Recall that imposing steady state in the top row of (16) defines ${\overline{Y}}$ from ${\overline{Y}}=\kappa DB{\overline{z}}^{\alpha -1}\left[ \phi {\overline{z}} {\overline{Y}}+{\underline{z}}-{\overline{z}}\right] ^{\rho }$, which shows that ${\overline{Y}}$ is increasing in *B*. Setting $B=\widehat{{\widehat{B}}}$ and ${\overline{z}}{\overline{Y}}={\overline{X}}$ gives

79 $$\begin{aligned} \widehat{{\widehat{B}}}=\frac{1}{{\overline{z}}}\left[ \frac{{\overline{X}}}{ \kappa D\left( \phi {\overline{X}}+{\underline{z}}-{\overline{z}}\right) ^{\rho }} \right] ^{\frac{1}{\alpha }}=\frac{1}{{\overline{z}}}\left[ \frac{{\overline{X}} }{\kappa D\left[ \phi {\underline{X}}\left( \frac{{\overline{z}}}{{\underline{z}}} \right) \right] ^{\rho }}\right] ^{\frac{1}{\alpha }}>0, \end{aligned}$$

where the second equality follows from (12) and (13). Since ${\overline{Y}}$ is increasing in *B*, it follows that $B>\widehat{{\widehat{B}}}$ is equivalent to ${\overline{z}}{\overline{Y}}>{\overline{X}}$.

(c) Using (78) and (79), and letting things cancel, we can write $\widehat{{\widehat{B}}}<{\widehat{B}}$ as

80 $$\begin{aligned} \left( \frac{{\underline{z}}}{{\overline{z}}}\right) ^{\rho +\alpha }=\left( \frac{{\underline{z}}}{{\overline{z}}}\right) ^{1+\frac{\alpha }{\sigma }}<\frac{ {\underline{X}}}{{\overline{X}}}. \end{aligned}$$

where the equality recalls $\rho =(\alpha /\sigma )+1-\alpha$ from (46). Next, using (12) and (13), and dividing both sides by ${\underline{z}}/{\overline{z}}$, we can write the inequality in (80) as

81 $$\begin{aligned} \left( \frac{{\underline{z}}}{{\overline{z}}}\right) ^{\frac{\alpha }{\sigma }}< \frac{{\underline{X}}/{\underline{z}}}{{\overline{X}}/{\overline{z}}}=\frac{\sigma (1-\alpha \beta )+\alpha \beta }{\left[ \beta \sigma (1-\alpha )+\sigma +\alpha \beta \right] -\beta \sigma \left( \frac{{\underline{z}}}{{\overline{z}}} \right) }, \end{aligned}$$

which expresses the condition for $\widehat{{\widehat{B}}}<{\widehat{B}}$ in terms of the ratio ${\underline{z}}/{\overline{z}}<1$. Letting ${\underline{z}}$ go to zero (keeping ${\overline{z}}$ constant), the right-hand side of (81) goes to something strictly positive, while the left-hand side goes to zero. Thus, the inequality in (81) must hold for ${\underline{z}}$ sufficiently close to zero, implying in turn that $\widehat{{\widehat{B}}}< {\widehat{B}}$ holds for ${\underline{z}}$ sufficiently close to zero.

(d) Part (i): The result follows from (14). The bottom row equals ${\underline{\tau }}=[\sigma (1-\beta )+\alpha \beta ]/[\sigma (1-\alpha \beta )+\alpha \beta ]$, and ${\overline{\tau }}$ is defined as the top row, evaluated at $z_{t}Y_{t}={\overline{z}}{\overline{Y}}\,$, which can be written:

82 $$\begin{aligned} {\overline{\tau }}=1-(1-{\underline{\tau }})\left[ 1-\left( \frac{{\overline{z}}- {\underline{z}}}{\phi }\right) \frac{1}{{\overline{z}}{\overline{Y}}}\right] = {\underline{\tau }}+\left( \frac{{\overline{z}}-{\underline{z}}}{\phi }\right) \frac{1-{\underline{\tau }}}{{\overline{z}}{\overline{Y}}}>\underline{\tau }\text {. } \end{aligned}$$

Part (ii): Given $B\in (\widehat{{\widehat{B}}},{\widehat{B}})$, and the way we defined $\widehat{{\widehat{B}}}$ and ${\widehat{B}}$ in the proof of (a) and (b), we know that the output levels in the two steady states, ${\overline{Y}}$ and ${\underline{Y}}$, must be such that ${\overline{Y}}>{\overline{X}}/{\overline{z}}$ (since $B>\widehat{{\widehat{B}}}$) and ${\underline{Y}}<{\underline{X}}/ {\underline{z}}$ (since $B<{\widehat{B}}$). From (12) and (13) it follows that

83 $$\begin{aligned} \frac{{\overline{X}}}{{\overline{z}}}-\left( \frac{{\overline{z}}-{\underline{z}}}{ {\overline{z}}\phi }\right) =\frac{{\underline{X}}}{{\underline{z}}}, \end{aligned}$$

implying that ${\overline{Y}}>{\overline{X}}/{\overline{z}}>{\underline{X}}/ {\underline{z}}>{\underline{Y}}$ (since ${\overline{z}}>{\underline{z}}$).

Part (iii): Using (5) the population levels in the two steady states can be written ${\overline{L}}=\gamma (1-\overline{\tau }){\overline{Y}}$ and ${\underline{L}}=\gamma (1-\underline{\tau }){\underline{Y}}$, respectively. From (31) follows that

84 $$\begin{aligned} {\overline{L}}=\gamma (1-{\overline{\tau }}){\overline{Y}}>\gamma \left[ \frac{ \beta \sigma (1-\alpha )}{\sigma (1-\alpha \beta )+\alpha \beta }\right] \left[ \frac{{\overline{X}}}{{\overline{z}}}-\left( \frac{{\overline{z}}- {\underline{z}}}{{\overline{z}}\phi }\right) \right] , \end{aligned}$$

where we have used $x_{t}=({\overline{z}}-{\underline{z}})/\phi$ and ${\overline{Y}}>{\overline{X}}/{\overline{z}}$; recall that $x_{t}=({\overline{z}}-{\underline{z}} )/\phi$ when ${\overline{z}}{\overline{Y}}>{\overline{X}}$, i.e., when $z_{t+1}\le {\overline{z}}$ binds. Using (31) again, we see that

85 $$\begin{aligned} {\underline{L}}=\gamma (1-{\underline{\tau }}){\underline{Y}}<\gamma \left[ \frac{ \beta \sigma (1-\alpha )}{\sigma (1-\alpha \beta )+\alpha \beta }\right] \frac{{\underline{X}}}{{\underline{z}}}, \end{aligned}$$

where we have used $x_{t}=0$ and ${\underline{Y}}<{\underline{X}}/{\underline{z}}$ ; recall that $x_{t}=0$ when ${\underline{Y}}<{\underline{X}}/{\underline{z}}$, i.e., when $z_{t+1}\ge {\underline{z}}$ binds. Now (83), (84 ), and (85) together imply that ${\overline{L}}>{\underline{L}}$. $\square$

### Proof of Proposition 2

The expression for ${\widetilde{Y}}$ follows from imposing steady state on ( 20). The expression for ${\widetilde{\tau }}$ in (21) can be found by solving (57) and (58) for $\tau _{t}$. The expression for ${\widetilde{\tau }}$ is identical to that in the bottom row in (14 ), which was derived by setting $x_{t}=0$ in (31) in the benchmark setting. $\square$

### Proof of Proposition 3

(a) Solving for ${\underline{Y}}$ from the bottom row in (76) gives

86 $$\begin{aligned} {\underline{Y}}=B\left[ \kappa D\phi ^{1-\alpha }\right] ^{\frac{1}{\alpha }} . \end{aligned}$$

Let $B^{*}$ be defined as the level of *B* that generates ${\underline{z}} {\underline{Y}}={\underline{X}}$. From (86) follows that

87 $$\begin{aligned} B^{*}=\frac{{\underline{X}}}{{\underline{z}}}\left[ \frac{1}{\kappa D\phi ^{1-\alpha }}\right] ^{\frac{1}{\alpha }}>0. \end{aligned}$$

From (86) we see that ${\underline{Y}}$ is increasing in *B*. By implication, if, and only if, $B<B^{*}$, then ${\underline{z}}{\underline{Y}} <{\underline{X}}$.

(b) The top row in (76) gives an implicit definition of ${\overline{Y}}$:

88 $$\begin{aligned} {\overline{Y}}=\kappa DB^{\alpha }{\overline{z}}^{\alpha -1}\left[ \phi {\overline{z}}{\overline{Y}}+{\underline{z}}-{\overline{z}}\right] ^{1-\alpha }\text { ,} \end{aligned}$$

which shows that $${\overline{Y}}$$ is increasing in *B*. Let $$B^{**}$$ be defined as the level of *B* that generates $${\overline{z}}{\overline{Y}}= {\overline{X}}$$. Setting $$B=B^{**}$$ and $${\overline{z}}{\overline{Y}}= {\overline{X}}$$ in (88) gives

89 $$\begin{aligned} B^{**}=\frac{1}{{\overline{z}}}\left[ \frac{{\overline{X}}}{\kappa D\left( \phi {\overline{X}}+{\underline{z}}-{\overline{z}}\right) ^{1-\alpha }} \right] ^{\frac{1}{\alpha }}=\frac{1}{{\overline{z}}}\left[ \frac{{\overline{X}} }{\kappa D\left[ \phi {\underline{X}}\left( \frac{{\overline{z}}}{{\underline{z}}} \right) \right] ^{1-\alpha }}\right] ^{\frac{1}{\alpha }}>0, \end{aligned}$$

where the second equality follows from (24) and (25). Since $${\overline{Y}}$$ is increasing in *B*, it follows that $$B>B^{**}$$ is equivalent to $${\overline{z}}{\overline{Y}}>{\overline{X}}$$.

(c) Using the expression for $$B^{**}$$ in (89), we see that

90 $$\begin{aligned} \begin{array}{cl} B^{**} &{} =\frac{1}{{\overline{z}}}\left[ \frac{{\overline{X}}}{\kappa D \left[ \phi {\underline{X}}\left( \frac{{\overline{z}}}{{\underline{z}}}\right) \right] ^{1-\alpha }}\right] ^{\frac{1}{\alpha }} \\ &{} =\frac{{\underline{X}}}{{\underline{z}}}\left[ \frac{\left( \frac{{\overline{X}} }{{\overline{z}}}\right) \left( \frac{{\underline{z}}}{{\underline{X}}}\right) }{ \kappa D\phi ^{1-\alpha }}\right] ^{\frac{1}{\alpha }} \\ &{} =B^{*}\left[ \left( \frac{{\overline{X}}}{{\overline{z}}}\right) \left( \frac{{\underline{z}}}{{\underline{X}}}\right) \right] ^{\frac{1}{\alpha }}. \end{array} \end{aligned}$$

where the last equality uses (87). From (24) and (25) we see that $${\overline{X}}/{\overline{z}}={\underline{X}}/{\underline{z}}+( {\overline{z}}-{\underline{z}})/(\phi {\overline{z}})>{\underline{X}}/{\underline{z}}$$ , which implies that the expression in square brackets following the last equality in (90) is greater than one. Thus, $$B^{**}>B^{*}$$.

(d) If the steady state $$(z^{\text {int}},Y^{\text {int}})$$ exists, it must be such that $$z^{\text {int}}Y^{\text {int}}\in ({\underline{X}},{\overline{X}})$$. This follows from the assumption $$B\in (B^{*},B^{**})$$, and parts (a) and (b) of the proposition: $$B>B^{*}$$ implies that $$z^{\text { int}}Y^{\text {int}}<{\underline{X}}$$ cannot hold; and $$B<B^{**}$$ implies that $$z^{\text {int}}Y^{\text {int}}>{\overline{X}}$$ cannot hold.

To show that the steady state $(z^{\text {int}},Y^{\text {int}})$ exists and is unique we derive closed-form expressions for $z^{\text {int}}$ and $Y^{ \text {int}}$. Consider the maximization problem in (22) and (23 ) for some given levels of $z_{t}$ and $Y_{t}$, such that $z_{t}Y_{t}\in ( {\underline{X}},{\overline{X}})$, meaning the solution to the maximization problem must be interior.

The first-order conditions for $x_{t}$ and $\tau _{t}$ in an interior solution can be written

91 $$\begin{aligned} \begin{array}{cl} (1-\beta )\left[ c_{t}^{R}\right] ^{-1} &{} =\phi \beta z_{t+1}^{-1}, \\ (1-\beta )\left[ c_{t}^{R}\right] ^{-1}z_{t}Y_{t} &{} =\beta (1-\alpha )(1-\tau _{t})^{-1}, \end{array} \end{aligned}$$

where we recall that ${\underline{z}}+\phi x_{t}=z_{t+1}$ in an interior solution. Together the conditions in (91) give

92 $$\begin{aligned} z_{t+1}=\left( \frac{\phi }{1-\alpha }\right) (1-\tau _{t})z_{t}Y_{t}. \end{aligned}$$

Imposing steady state, and using super-index “int” to denote steady-state levels, we can now write:

93 $$\begin{aligned} \begin{array}{cl} (1-\tau ^{\text {int}})Y^{\text {int}} &{} =\frac{1-\alpha }{\phi }, \\ L^{\text {int}} &{} =\gamma (1-\tau ^{\text {int}})Y^{\text {int}}, \\ Y^{\text {int}} &{} =B^{\alpha }\left( L^{\text {int}}\right) ^{1-\alpha }\text {, } \end{array} \end{aligned}$$

where the top row imposes steady-state on (92), and the middle and bottom rows do the same for $L_{t+1}=\gamma (1-\tau _{t})Y_{t}$ and $Y_{t}=B^{\alpha }L_{t}^{1-\alpha }$, respectively; see (23). Solving (93) for $L^{\text {int}}$, $Y^{\text {int}}$ and $\tau ^{\text {int}}$ we get

94 $$\begin{aligned} \begin{array}{cl} L^{\text {int}} &{} =\frac{\gamma \left( 1-\alpha \right) }{\phi }, \\ Y^{\text {int}} &{} =B^{\alpha }\left[ \frac{\gamma \left( 1-\alpha \right) }{ \phi }\right] ^{1-\alpha }, \\ \tau ^{\text {int}} &{} =1-\left( \frac{1}{\gamma }\right) ^{1-\alpha }\left( \frac{1-\alpha }{B\phi }\right) ^{\alpha }. \end{array} \end{aligned}$$

Finally, we derive an expression for the steady-state level of $z_{t}$, denoted $z^{\text {int}}$. To that end, we first rewrite the first-order condition for $x_{t}$ in (91) as

95 $$\begin{aligned} (1-\beta )\left[ \tau _{t}z_{t}Y_{t}-\left( \frac{z_{t+1}-{\underline{z}}}{ \phi }\right) \right] ^{-1}=\phi \beta z_{t+1}^{-1}, \end{aligned}$$

where we have used $c_{t}^{R}=\tau _{t}z_{t}Y_{t}-x_{t}$ and $z_{t+1}= {\underline{z}}+\phi x_{t}$, implying $x_{t}=\left( z_{t+1}-{\underline{z}} \right) /\phi$; recall (23) again. Rearranging (95), and imposing steady state, gives us $z^{\text {int}}$ in terms of $\tau ^{\text { int}}Y^{\text {int}}$:

96 $$\begin{aligned} z^{\text {int}}=\frac{\beta {\underline{z}}}{1-\beta \phi \tau ^{\text {int}}Y^{ \text {int}}}. \end{aligned}$$

Next we can use (94) to find that

97 $$\begin{aligned} \begin{array}{cl} \beta \phi \tau ^{\text {int}}Y^{\text {int}} &{} =\beta \phi \left( B^{\alpha } \left[ \frac{\gamma \left( 1-\alpha \right) }{\phi }\right] ^{1-\alpha }- \frac{1-\alpha }{\phi }\right) \\ &{} =\beta \left( \phi B\right) ^{\alpha }\left[ \gamma \left( 1-\alpha \right) \right] ^{1-\alpha }-\beta \left( 1-\alpha \right) \end{array} \end{aligned}$$

Substituting (97) into (96) gives

98 $$\begin{aligned} z^{\text {int}}=\frac{\beta {\underline{z}}}{1+\beta (1-\alpha )-\beta \left( \phi B\right) ^{\alpha }\left[ \gamma \left( 1-\alpha \right) \right] ^{1-\alpha }}. \end{aligned}$$

The existence and uniqueness of the steady state is shown by the closed-form expressions for $$Y^{\text {int}}$$ and $$z^{\text {int}}$$ in (94) and (98). One can also use (94) and (98) to verify that $$z^{\text {int}}\in ({\underline{z}},{\overline{z}})$$ and $$z^{\text {int}}Y^{\text { int}}\in ({\underline{X}},{\overline{X}})$$ when $$B\in (B^{*},B^{**})$$.

The claims in (i)–(iv) are confirmed by differentiating the expressions in (94) and (98) with respect to *B* and $\phi$. $\square$

## Empirics

### Simulation

Let $Y_{i,t}$ and $z_{i,t}$ be output and extractive capacity, respectively, of society *i* in period *t*. The simulation is done by iterating on (15) and (16), given some initial values for $Y_{i,t}$ and $z_{i,t}$ , with $\phi$, *D*, ${\underline{X}}$, and ${\overline{X}}$ replaced by $\phi _{t}$, $D_{t}$, ${\underline{X}}_{t}$, and ${\overline{X}}_{t}$ (the time-dependent levels of the same variables), and with *B* replaced by $B_{i}$ (the society-specific level of *B*). Compactly, this can be written as

99 $$\begin{aligned} \begin{array}{ll} z_{i,t+1}= &{} \Phi (Y_{i,t},z_{i,t};\phi _{t},D_{t},{\underline{X}}_{t}, {\overline{X}}_{t}), \\ Y_{i,t+1}= &{} \Psi (Y_{i,t},z_{i,t},B_{i};\phi _{t},D_{t},{\underline{X}}_{t}, {\overline{X}}_{t}), \end{array} \end{aligned}$$

where $D_{t}$, ${\underline{X}}_{t}$, and ${\overline{X}}_{t}$ are given by (47), (12), and (13), with $\phi _{t}$ replacing $\phi$, and where the functions $\Phi$ and $\Psi$ are defined in (15) and (16). To determine $\phi _{t}$, first let

100 $$\begin{aligned} z_{t}^{\text {mean}}=\frac{1}{200}\sum \limits _{i=1}^{200}z_{i,t} \end{aligned}$$

denote the mean of $z_{i,t}$ in period *t* across the 200 societies. We then let $\phi _{t}$ depend on $z_{t}^{\text {mean}}$ according to

101 $$\begin{aligned} \phi _{t}=\left[ 1-\left( \frac{z_{t}^{\text {mean}}-{\underline{z}}}{\overline{ z}-{\underline{z}}}\right) \right] \phi _{0}+\left( \frac{z_{t}^{\text {mean}}- {\underline{z}}}{{\overline{z}}-{\underline{z}}}\right) \times 30\phi _{0}, \end{aligned}$$

where $\phi _{0}$ is the exogenously given initial value for $\phi _{t}$. Note that $z_{t}^{\text {mean}}\in [{\underline{z}},{\overline{z}}]$ and that the weight $(z_{t}^{\text {mean}}-{\underline{z}})/({\overline{z}}- {\underline{z}})$ increases from zero to one as $z_{t}^{\text {mean}}$ goes from ${\underline{z}}$ to ${\overline{z}}$, implying an increase in $\phi _{t}$ by a factor of 30, which is sufficient to ensure that all 200 societies make a full transition.

The values of $B_{i}$ are uniformly distributed on the interval $(\widehat{ {\widehat{B}}},{\widehat{B}})$, given by (78) and (79), with $\phi$ , *D*, ${\underline{X}}$, and ${\overline{X}}$ replaced by $\phi _{0}$, $D_{0}$, ${\underline{X}}_{0}$, and ${\overline{X}}_{0}$ (see above). That is, $B_{1}=\widehat{{\widehat{B}}}$ and $B_{200}={\widehat{B}}$. This implies that all societies exhibit multiple steady states for fixed $\phi _{0}$.

Parameter values are set to $\alpha =.5$, $\sigma =2$, $\beta =.95$, $\eta =1$, ${\underline{z}}=.01$, and ${\overline{z}}=.99$. We set $\gamma$ to approximately 96.81, targeting $D_{0}$ to 100.

The initial value for $\phi _{t}$ is set to $\phi _{0}=.01$, which together with the parameter values above ensures that $\widehat{{\widehat{B}}}<{\widehat{B}}$.

Initial values for $z_{i,t}$ are set at the minimum level, $z_{i,0}= {\underline{z}}=.01$, for all *i*. Initial levels of $Y_{i,t}$ for each society are set at the low-extractive steady-state values associated with their respective $B_{i}$, i.e., $Y_{i,0}=\left[ \kappa D_{0}B_{i}^{\alpha } {\underline{z}}^{\alpha -1}\left( \phi _{0}{\underline{z}}\right) ^{\rho }\right] ^{1/(1-\rho )}$; see (17).

Since all societies are dropped off in the low-extractive steady-state they stay there until an exogenous shock is introduced at $t=40$. At that point, the levels of $z_{i,t}$ increase from ${\underline{z}}=.01$ to ${\overline{z}} =.99$ for every tenth society when ranked by $B_{i}$ (the first being $i=10$ and the last $i=191$). From that point on, all societies follow the dynamical process described be (99) to (101), eventually transitioning to the high-extractive steady state.

### Data

The measure of statehood is from Borcan et al. (2018), in turn building on Bockstette et al. (2002). They report a score on the extent of statehood across territories defined by modern countries and by half century, from 3500 BCE until today. This index is based on three different criteria: whether any government above the tribal level was present; whether this government was local or foreign; and how much of the territory of the modern country that was controlled by the government. Here we use the accumulated state index score from 3500 BCE to to 1500 CE or 450 CE. Both endpoints precede European colonization and the change in crop composition following the Columbian exchange.

Countries without statehood before 450 CE and 1000 BCE, respectively, are defined as those with zero state index score from 3500 BCE to that point in time.

Land productivity is measured by the Caloric Suitability Index, which is from Galor and Özak (2016) and available here:

https://ozak.github.io/Caloric-Suitability-Index/

Specifically, we use the country-level measure of mean productivity across crops and locations in a country, excluding non-productive locations, and using only crops available before 1500 CE.

Distances to state origin are obtained by applying the geodist package in Stata to calculate the distance from the centroids of modern country borders to the geo-coordinates of Baghdad and Beijing, respectively. Distance to state origin is the shortest of those two distances.

To measure country borders we use publicly available shapefiles shared through the ESRI/ArcGIS website, downloadable here:

https://www.arcgis.com/home/item.html?id=2ca75003ef9d477fb22db19832c9554f

Latitude and regional dummies are from the same data. Latitude refers to the country centroid.

## Footnotes

1 For example, Hibbs and Olsson (2004, p. 3718) write that “[t]he superior agricultural mode of production made possible specialization of economic activity and the establishment of a non-food producing class devoted to the creation and codification of knowledge and the development of technology.” Diamond (1997, p. 285) writes that “food production [i.e., agriculture] may be organized so as to generate stored food surpluses, which permit economic specialization and social stratification.” In Mann (1986), an oft-cited overview of the literature on early state development, the index lists 26 pages referencing the term “surplus” in various contexts.

2 Dal Bó et al. (2016) capture the interaction between what we may call productive and defensive capacities, while we here focus on productive and extractive capacities. Conceptually, extractive capacity may here represent the powers of a domestic ruler to tax his own people. By contrast, defensive capacity would rather capture the ability to protect against extraction by external and less benevolent actors.

3 Besley et al. (2013) set up a dynamic, but non-Malthusian, model of investment in state capacity.

4 In that case, the ruling collective is assumed to be cohesive enough to act as one agent. It also carries fixed size, meaning each member has one offspring, replacing the (single) parent in the next period.

5 The effective tax base may correspond to what Scott (2009, p. 73) has called “state-accessible product.”

6 We could also let $A_{t}$ include external defense, which is a type of public good. See Sect. 3.6.

7 One could imagine other interpretations too. Following Carneiro (1970), one may also think of $\phi$ as capturing the degree of environmental circumscription. For example, creating records over tax payers may be easier when their ability to move is limited.

8 This utility function is chosen for tractability. Another approach would be a dynastic model where the ruler cares about the utility of the next generation. Letting $V(z_{t},Y_{t})$ be the ruler’s value function, the associated Bellman equation could then be written $V(z_{t},Y_{t})=\max {{\ln \left( c_{t}^{R}\right) +\beta V(z_{t+1},Y_{t+1})}}$, subject to the budget constraints in (11) below.

9 That is, one can let the production function in (1) be written $Y_{t}=(B\left[ A_{t}+{\underline{A}}\right] )^{\alpha }L_{t}^{1-\alpha }$, where ${\underline{A}}$ is an exogenous lower bound for productive capacity.

10 One element that the extended model in the Online Appendices does not capture is an endogenous decision by the potential predator, which can generate a link from output to the probability of theft. For such a model, see Dal Bó et al. (2016).

11 Note that $Y_{0}$ and $z_{0}$ are exogenously given, so nothing forces the economy to end up on that saddle path. Put another way, if $Y_{0}$ and $z_{0}$ were drawn from a joint continuous distribution, then the economy would end up on the saddle path with zero probability.

12 That is, ${\underline{\tau }}$ is given by the bottom row in (14), which does not depend on ${\underline{z}}$.

13 While the (gross) extraction rate is a constant ${\widetilde{\tau }}$, following Mayshar et al. (2020) we may instead consider the *net* extraction rate. This is the same as the rate of extraction, ${\widetilde{\tau }}$, minus the (implicit) cost of extraction, $(1-{\widetilde{z}}) \widetilde{\tau }$; cf. Sect. 3.6.5. The net extraction rate here equals just ${\widetilde{\tau }}-(1-{\widetilde{z}}){\widetilde{\tau }}= {\widetilde{z}}{\widetilde{\tau }}$, which is increasing in ${\widetilde{z}}$ (since ${\widetilde{\tau }}$ does not depend on ${\widetilde{z}}$). This is consistent with Proposition B2 in Mayshar et al. (2020, Online Appendices B).

14 It can be seen that $$B^{*}$$ and $$B^{**}$$ coincide with the corresponding expressions in the benchmark setting, $${\widehat{B}}$$ and $$\widehat{{\widehat{B}}}$$, when $$\sigma$$ goes to infinity. That is, $$\lim _{\sigma \rightarrow \infty }{\widehat{B}}=B^{*}$$ and $$\lim _{\sigma \rightarrow \infty }\widehat{{\widehat{B}}}=B^{**}$$.

15 One way to see this more formally is to note that the two thresholds for *B* , above which the high-extractive steady-state exists and the low-extractive one does not, are both decreasing in $\phi$. These are the ones denoted $\widehat{{\widehat{B}}}$ and ${\widehat{B}}$, respectively, in Proposition 1.

16 The small dip in mean extractive capacity for those with the lowest levels of *B* is due to $z_{t}$ temporarily falling below ${\overline{z}}$ in the transition to the high-extractive steady state.

## References

[CR1] Allen RC (1997). Agriculture and the origins of the state in ancient Egypt. Explorations in Economic History.

[CR2] Ashraf Q, Galor O (2011). Dynamics and stagnation in the Malthusian epoch. American Economic Review.

[CR3] Besley T, Persson T (2009). The origins of state capacity: Property rights, taxation, and politics. American Economic Review.

[CR4] Besley T, Persson T (2011). Pillars of prosperity: The political economics of development clusters.

[CR5] Besley T, Ilzetzki E, Persson T (2013). Weak states and steady states: The dynamics of fiscal capacity. American Economic Journal: Macroeconomics.

[CR6] Bockstette V, Chanda A, Putterman L (2002). States and markets: The advantage of an early start. Journal of Economic Growth.

[CR7] Borcan O, Olsson O, Putterman L (2018). State history and economic development: Evidence from six millennia. Journal of Economic Growth.

[CR8] Borcan, O., Olsson, O., & Putterman, L. (2020). Transition to agriculture and first state presence: A global analysis. University of Gothenburg working paper in economics no. 741.

[CR9] Carneiro RL (1970). A theory of the origin of the state. Science.

[CR10] Chanda A, Putterman L (2007). Early starts, reversals and catch-up in the process of economic development. Scandinavian Journal of Economics.

[CR11] Chanda A, Cook CJ, Putterman L (2014). Persistence of fortune: Accounting for population movements, there was no post-Columbian reversal. American Economic Journal: Macroeconomics.

[CR12] Childe, V. G. (1936). Man makes himself. The New American Library, Inc. (Reprinted 1951).

[CR13] Childe VG (1950). The urban revolution. The Town Planning Review.

[CR14] Dal Bó, E., Hernández, P., Mazzuca, S. (2016). The paradox of civilization: pre-institutional sources of security and prosperity, mimeo, UC Berkeley, NYU Abu Dabi, and Johns Hopkins.

[CR15] Depetris-Chauvin, E., (2016). State history and contemporary conflict: Evidence from Sub-Saharan Africa, mimeo, Pontificia Universidad Cat ólica de Chile.

[CR16] Depetris-Chauvin, E., & Özak, Ö. (2016) Population diversity, division of labor and comparative development, mimeo, Pontificia Universidad Católica de Chile and Southern Methodist University.

[CR17] Diamond J (1997). Guns, germs, and steel: The fates of human societies.

[CR18] Ertman T (1997). Birth of the leviathan: Building states and regimes in medieval and early modern Europe.

[CR19] Fenske J (2014). Ecology, trade, and states in pre-colonial Africa. Journal of the European Economic Association.

[CR20] Flannery KV (1972). The cultural evolution of civilizations. Annual Review of Ecology and Systematics.

[CR21] Galor O (2010). The 2008 Lawrence R. Klein lecture-comparative economic development: Insights from Unified Growth Theory. International Economic Review.

[CR22] Galor O, Özak Ö (2016). The agricultural origins of time preference. American Economic Review.

[CR23] Hariri JG (2012). The autocratic legacy of early statehood. American Political Science Review.

[CR24] Heldring, L., Allen, R. C., & Bertazzin, M. C. (2019). Institutional adaptation to environmental change, mimeo, Institute on Behavior & Inequality (briq), Bonn.

[CR25] Hibbs DR, Olsson O (2004). Geography, biogeography, and why some countries are rich and others are poor. Proceedings of the National Academy of Sciences.

[CR26] Lagerlöf N-P (2016). Statehood, democracy and preindustrial development. Journal of Economic Dynamics and Control.

[CR27] Lindstrom L (1981). “Big man:” A short terminological history. American Anthropologist.

[CR28] Litina, A. (2014). The geographical origins of early state formation, CREA Discussion Paper Series 14-28, Center for Research in Economic Analysis, University of Luxembourg.

[CR29] Mann M (1986). The sources of social power, volume 1: a history of power from the beginning to AD 1760.

[CR30] Mayoral, L., & Olsson, O. (2020). Pharaoh’s cage: Environmental circumscription and appropriability in early state development, mimeo, University of Gothenburg.

[CR31] Mayshar J, Moav O, Neeman Z (2017). Geography, transparency, and institutions. American Political Science Review.

[CR32] Mayshar, J., Moav, O., Neeman, Z., & Pascali, L. (2020). The origin of the state: Land productivity or appropriability? Mimeo, Hebrew University of Jerusalem, University of Warwick, Tel-Aviv University, and Pompeu Fabra University.

[CR33] Nissen HJ, Heine P (2009). From Mesopotamia to Iraq: A concise history.

[CR35] Putterman L (2008). Agriculture, diffusion and development: Ripple effects of the Neolithic Revolution. Economica.

[CR36] Read KE (1959). Leadership and consensus in a New Guinea society. American Anthropologist.

[CR37] Sahlins MD (1963). Poor man, rich man, big-man, chief: Political types in Melanesia and Polynesia. Comparative Studies in Society and History.

[CR38] Scott JC (2009). The art of not being governed: An anarchist history of Upland Southeast Asia.

[CR39] Scott JC (2017). Against the grain: A deep history of the earliest states.

[CR40] Service, E. R (1975). Origins of the state and civilization: The process of cultural evolution.

[CR41] Schönholzer D (2019). The origin of the state: Incentive compatible extraction under environmental circumscription, mimeo.

[CR42] Stasavage D (2020). The decline and rise of democracy: A global history from antiquity to today.

[CR43] Stevens Curl J, Wilson S (2015). A dictionary of architecture and landscape architecture.

[CR44] Wittfogel KA (1957). Oriental despotism: A comparative study of total power.

