# Supplementary material for: Multiple steady statehood: the roles of productive and extractive capacities
Source: J Econ Growth (Boston). 2021 Mar 13;26(2):113–52. doi: 10.1007/s10887-021-09188-9 (PMC8550320; doi:10.1007/s10887-021-09188-9)
Supplement: Supplementary file 1 — Supplementary material 1 (pdf 188 KB) [file 10887_2021_9188_MOESM1_ESM.pdf]

Online Appendix to Multiple Steady Statehood

This Online Appendix explores some alternative ways to set up and interpret the model in "Multiple Steady Statehood: The Roles of Productive and Extractive Capacities" by Nils-Petter Lagerlöf. Equations and propositions referenced can be found in the original paper, when not in these notes.

The model without investment in productive capacity: interior solutions

Consider the setting without investment in productive capacity under the parametric case where

$$B^* < B < B^{**}. \tag{A.1}$$

As in the paper, we denote the steady-state levels of z_t and Y_t under this parametric configuration by z^{int} and Y^{int}, respectively. From Proposition 3, we recall that no steady state can exist with $z^{\text{int}} = \underline{z}$ (since $B > B^*$) or $z^{\text{int}} = \overline{z}$ (since $B < B^{**}$). The steady state must therefore be such that $z^{\text{int}} \in (\underline{z}, \overline{z})$, and $z^{\text{int}} Y^{\text{int}} \in (\underline{X}, \overline{X})$.

The dynamics of z_t and Y_t when $z_t Y_t \in (\underline{X}, \overline{X})$ are given by (68) and (75), which we restate here:

$$z_{t+1} = \frac{\beta\left(\phi z_t Y_t + \underline{z}\right)}{1+\beta(1-\alpha)}, \tag{A.2}$$

$$Y_{t+1} = B^{\alpha}\left[\frac{\gamma\beta\left(1-\alpha\right)}{1+\beta\left(1-\alpha\right)}\right]^{1-\alpha}\left(\frac{\phi z_t Y_t + \underline{z}}{\phi z_t}\right)^{1-\alpha}. \tag{A.3}$$

Deriving z^{int} and Y^{int} again

We first verify that we can find the expressions for z^{int} and Y^{int} in (94) by imposing steady state on the dynamical system in (A.2) and (A.3). Setting $z_{t+1} = z_t = z^{\text{int}}$, and $Y_{t+1} = Y_t = Y^{\text{int}}$, we first note from (A.2) that

$$\frac{\phi z^{\text{int}} Y^{\text{int}} + \underline{z}}{z^{\text{int}}} = \frac{1+\beta(1-\alpha)}{\beta}. \tag{A.4}$$

Then (A.3) shows that

$$\begin{aligned} Y^{\text{int}} &= B^{\alpha}\left[\tfrac{\gamma\beta(1-\alpha)}{1+\beta(1-\alpha)}\right]^{1-\alpha}\left(\tfrac{\phi z^{\text{int}} Y^{\text{int}} + \underline{z}}{\phi z^{\text{int}}}\right)^{1-\alpha} \\ &= B^{\alpha}\left[\tfrac{\gamma\beta(1-\alpha)}{1+\beta(1-\alpha)}\right]^{1-\alpha}\left[\tfrac{1+\beta(1-\alpha)}{\phi\beta}\right]^{1-\alpha} \\ &= B^{\alpha}\left[\tfrac{\gamma(1-\alpha)}{\phi}\right]^{1-\alpha}, \end{aligned} \tag{A.5}$$

where the second equality uses (A.4), and which is identical to the expression for Y^{int} derived in (94).

Next (A.4) and (A.5) show that

$$\begin{aligned} z^{\text{int}} &= \frac{\beta \underline{z}}{1+\beta(1-\alpha)-\beta\phi Y^{\text{int}}} \\ &= \frac{\beta \underline{z}}{1+\beta(1-\alpha)-\beta\phi B^{\alpha}\left[\frac{\gamma(1-\alpha)}{\phi}\right]^{1-\alpha}} \\ &= \frac{\beta \underline{z}}{1+\beta(1-\alpha)-\beta(\phi B)^{\alpha}[\gamma(1-\alpha)]^{1-\alpha}}, \end{aligned} \tag{A.6}$$

which is the same as in (98).

Showing that $z^{\text{int}} \in (\underline{z}, \overline{z})$ and $z^{\text{int}}Y^{\text{int}} \in (\underline{X}, \overline{X})$

First we derive expressions for B^* and B^{**} in terms of exogenous variables only, not involving κ, D, $\underline{X}$, or $\overline{X}$. Recall from (87) that

$$B^* = \frac{\underline{X}}{\underline{z}}\left[\frac{1}{\kappa D \phi^{1-\alpha}}\right]^{\frac{1}{\alpha}}. \tag{A.7}$$

Using the expressions for κ an D in (77) we can write

$$\frac{1}{\kappa D \phi^{1-\alpha}} = \left[\frac{1-\alpha\beta}{\gamma\beta(1-\alpha)}\right]^{1-\alpha}. \tag{A.8}$$

Then recall from (25) that

$$\frac{\underline{X}}{\underline{z}} = \frac{1-\alpha\beta}{\beta\phi}. \tag{A.9}$$

Now substituting (A.8) and (A.9) into (A.7) allows us write B^* as

$$B^* = \frac{1}{\phi}\left(\frac{1-\alpha\beta}{\beta}\right)^{\frac{1}{\alpha}}\left[\frac{1}{\gamma(1-\alpha)}\right]^{\frac{1-\alpha}{\alpha}}. \tag{A.10}$$

Next recall from (25) that

$$B^{**} = B^*\left[\left(\frac{\overline{X}}{\overline{z}}\right)\left(\frac{\underline{z}}{\underline{X}}\right)\right]^{\frac{1}{\alpha}}, \tag{A.11}$$

and then use (60) to write

$$\frac{\overline{X}}{\overline{z}} = \frac{1}{\phi}\left[\frac{1+\beta(1-\alpha)}{\beta} - \frac{\underline{z}}{\overline{z}}\right]. \tag{A.12}$$

Now substituting (A.9) and (A.12) into (A.11) shows that

$$\begin{aligned} B^{**} &= B^{*}\left(\tfrac{\beta}{1-\alpha\beta}\right)^{\frac{1}{\alpha}}\left[\tfrac{1+\beta(1-\alpha)-\beta(\underline{z}/\overline{z})}{\beta}\right]^{\frac{1}{\alpha}} \\ &= \tfrac{1}{\phi}\left[\tfrac{1}{\gamma(1-\alpha)}\right]^{\frac{1-\alpha}{\alpha}}\left[\tfrac{1+\beta(1-\alpha)-\beta(\underline{z}/\overline{z})}{\beta}\right]^{\frac{1}{\alpha}}, \end{aligned} \tag{A.13}$$

where the last equality uses (A.7).

We can now use the expression for z^{int} in (98) to verify that setting $z^{\text{int}} = \underline{z}$ and solving for B gives the expression for B^* in (A.10). Since z^{int} is increasing in B this means that $z^{\text{int}} > \underline{z}$ for all $B > B^*$.

Analogously, we can set $z^{\text{int}} = \overline{z}$ in (98) and solve for B, which gives the expression for B^{**} in (A.13). This verifies that $z^{\text{int}} < \overline{z}$ for all $B < B^{**}$, since z^{int} is increasing in B. That is, $B \in (B^*, B^{**})$ implies $z^{\text{int}} \in (\underline{z}, \overline{z})$.

It now also follows that $z^{\text{int}}Y^{\text{int}} \in (\underline{X}, \overline{X})$ whenever $B \in (B^*, B^{**})$. To see this, note that $z^{\text{int}}Y^{\text{int}} < \underline{X}$ would imply that the constraint $z_t \geq \underline{z}$ is binding in steady state, contradicting $z^{\text{int}} > \underline{z}$. Similarly, $z^{\text{int}}Y^{\text{int}} > \overline{X}$ would imply that $z_t \leq \overline{z}$ binds in steady state, contradicting $z^{\text{int}} < \overline{z}$.

Stability

To explore (local) stability we can first define

$$W_t = \phi z_t Y_t + \underline{z}, \tag{A.14}$$

$$R = \frac{\beta}{1+\beta(1-\alpha)}, \tag{A.15}$$

and

$$Q = (\phi B)^{\alpha}\left[\gamma(1-\alpha)\right]^{1-\alpha}, \tag{A.16}$$

allowing us to write the dynamical system in (A.2) and (A.3) as

$$\begin{aligned} z_{t+1} &= RW_t, \\ W_{t+1} &= Qz_t^{\alpha-1}(RW_t)^{2-\alpha} + \underline{z}. \end{aligned} \tag{A.17}$$

If the system in (A.17) is locally stable, then that in (A.2) and (A.3) must also be.

The quickest way to determine the stability properties of (A.17) is to draw a phase diagram with z_t on the vertical axis and W_t on the horizontal axis.

The locus along which z_t is stationary ($z_{t+1} = z_t$) is a straight line with slope $R = \beta/[1+\beta(1-\alpha)]$, starting at the origin; $z_{t+1} > (<) z_t$ at coordinates below (above) the locus.

The corresponding locus along which W_t is stationary is given by setting $W_{t+1} = W_t$ in (A.17). This gives

$$z_t = Q^{\frac{1}{1-\alpha}} \left[\frac{(RW_t)^{2-\alpha}}{W_t - \underline{z}} \right]^{\frac{1}{1-\alpha}} \equiv \mathcal{L}^W(W_t), \tag{A.18}$$

where (A.17) verifies that $W_{t+1} < (>) W_t$ when $z_t > (<) \mathcal{L}^W(W_t)$.

We see right away that $\lim_{W_t \to \underline{z}} \mathcal{L}^W(W_t) = \infty$. Some algebra shows also that $\mathcal{L}^W(W_t)$ reaches a minimum at $W_t = W^{\min}$, given by

$$W^{\min} = \left(\frac{2-\alpha}{1-\alpha} \right) \underline{z},$$

implying that $\partial \mathcal{L}^W(W_t)/\partial W_t < (>) 0$ for $W_t < (>) W^{\min}$.

We can now illustrate the dynamics in a phase diagram with z_t on the vertical axis and W_t on the horizontal axis (left for the reader to draw). The $(z_{t+1} = z_t)$-locus is a straight line starting in the origin with slope $R = \beta/[1 + \beta(1-\alpha)] > 0$. The $(W_{t+1} = W_t)$-locus is given by $\mathcal{L}^W(W_t)$ in (A.18), and has negative slope for $W_t \in (\underline{z}, W^{\min})$. Thus, the two loci intersect along the downward-sloping segment of the $(W_{t+1} = W_t)$-locus, and it follows that the unique steady state must be given by that intersection.

[One can also show that there is no intersection along the upward-sloping segment, as long as $B < B^{**}$, and even if the two loci did intersect there, that intersection could not give the steady state equilibrium, since it would imply that z^{int} is decreasing in B, contradicting (A.6).]

The two loci separate the phase diagram into four regions surrounding the steady state. Using the information derived above about how z_t and W_t evolve over time off these loci, it can be seen that an economy starting off in any of the four regions will converge to the steady state. That is, any initial level of z_t and W_t is associated with a (unique) trajectory leading to the (unique) steady state, demonstrating stability.

Tax collectors: micro foundations for accumulation of extractive capacity

This section proposes one way to motivate the functional form for accumulation of extractive capacity, as given by (6) in the paper, i.e.,

$$z_{t+1} = \min\{\overline{z}, \underline{z} + \phi x_t\}. \tag{A.19}$$

To that end, we introduce a new group of agents, called *tax collectors*. These are tasked by the ruler with collecting $\tau_t Y_t$ in taxes from the subjects. After collecting the taxes the tax collectors have the option to run off with the revenue to a region where it is harder for the

ruler to reach. This can thus be thought of as a spatial model, where the ruler has full power at the centre and imperfect controls over what happens in the periphery.

If the tax collectors run away, we assume that a fraction $\delta \in [0, 1]$ of the revenue is lost, which captures the loss of grains and other in-kind revenue when transporting them.

The ruler has two ways to address the problem of tax collectors running away. First, he can build capacity to retrieve stolen revenue. Let r_t denote the fraction of the stolen revenue that can be retrieved. We assume that this capacity depends on resources spent by the ruler in the previous period, according to this functional form:

$$r_t = \min\{\overline{r}, \widetilde{\phi} x_{t-1}\}, \tag{A.20}$$

where x_{t-1} denotes resources spent in the previous period, $\widetilde{\phi} > 0$ is a parameter measuring how easy it is to build capacity to retrieve lost taxes, and where $\overline{r} \leq 1$ is an exogenous maximum level of r_t.

The second way the ruler can address the problem of tax collectors running away is to commit to a contract allowing tax collectors to keep some fraction of the revenue if they do not run away. Let that fraction be $1 - z_t$, meaning the ruler gets a fraction z_t.

The payoff to the tax collectors if they choose to stay thus equals $(1 - z_t)\tau_t Y_t$. That is, the tax collectors collect $\tau_t Y_t$ and keep a fraction $1 - z_t$. The corresponding payoff if the tax collectors run away with the revenue equals $(1 - r_t)(1 - \delta)\tau_t Y_t$. That is, $(1 - \delta)\tau_t Y_t$ is left of the stolen revenue after transporting it, and a fraction $1 - r_t$ of that is left after the ruler has retrieved the share r_t. For tax collectors not to run away it must thus hold that

$$(1 - r_t)(1 - \delta)\tau_t Y_t \leq (1 - z_t)\tau_t Y_t. \tag{A.21}$$

The ruler sets the contract so that his share of the revenue, z_t, is as high as possible, subject to (A.21). This means that (A.21) holds with equality, which gives us the ruler's share as

$$\begin{aligned} z_t &= 1 - (1 - r_t)(1 - \delta) \\ &= \delta + (1 - \delta) r_t \\ &= \delta + (1 - \delta) \min\{\overline{r}, \widetilde{\phi} x_{t-1}\} \\ &= \min\left\{\delta + (1 - \delta)\overline{r}, \delta + (1 - \delta)\widetilde{\phi} x_{t-1}\right\}, \end{aligned} \tag{A.22}$$

where the third equality uses (A.20). We can now define

$$\begin{aligned} \overline{z} &= \delta + (1 - \delta)\overline{r}, \\ \underline{z} &= \delta, \\ \phi &= (1 - \delta)\widetilde{\phi}, \end{aligned} \tag{A.23}$$

to rewrite the bottom row in (A.22) as

$$z_t = \min\{\overline{z}, \underline{z} + \phi x_{t-1}\}. \tag{A.24}$$

Forwarding (A.24) one period we get the same expression as in (A.19).

Intuitively, extractive capacity is measured by the ruler's negotiating position relative to the tax collectors. A higher x_t implies a higher r_{t+1}, and thus a higher z_{t+1}, because a ruler who can retrieve more of any stolen resources is able to impose a contract more favorable to himself.

Similarly, a higher $\overline{r}$ implies a higher $\overline{z}$, since the maximum amount that can be retrieved determines the maximum share the ruler can get in an incentive-compatible contract. The ruler is guaranteed a share of $\underline{z} = \delta$, since any contract that gives the tax collectors a share $1-\delta$ will induce them not to run away.

A low δ is associated with a high ϕ. Intuitively, the effects of a low δ is mitigated by investments in r_t.

ϕ also depends on $\widetilde{\phi}$, which may capture other factors which determine the return to investing in extractive capacity, e.g., how circumscribed the environment is.

Defense

The benchmark model can be extended to allow for investment in external defense, aside from other types of public goods. Suppose that some fraction of the output is subject to theft by external forces, and that the ruler can protect himself against such theft by undertaking costly investments. Let $1-P_{t+1}$ denote the fraction of output that is stolen by outside forces in period $t+1$, where $P_{t+1} \in [0,1]$. (In other words, P_{t+1} is the share of output that is "protected.") Output net of external theft is denoted by $\widetilde{Y}_{t+1}$, and given by

$$\widetilde{Y}_{t+1} = P_{t+1}Y_{t+1} = P_{t+1}(BA_{t+1})^{\alpha}L_{t+1}^{1-\alpha}. \tag{A.25}$$

The ruler can invest in P_{t+1}. This investment is undertaken in the preceding period at cost

$$\eta_P P_{t+1}^{\sigma_P}, \tag{A.26}$$

where $\eta_P > 0$ and $\sigma_P > 1$ are exogenous parameters of the external defense cost function. Analogously, we now denote the cost of investing in other public goods, A_{t+1}, by

$$\eta_A A_{t+1}^{\sigma_A}, \tag{A.27}$$

where $\eta_A > 0$ and $\sigma_A > 1$ are here the exogenous parameters characterizing the cost function for other public goods than defense.

The ruler's utility maximization problem can now be written:

$$\max_{\tau_t, x_t, A_{t+1}} (1-\beta)\ln\left(c_t^R\right) + \beta\ln(z_{t+1}\widetilde{Y}_{t+1}), \tag{A.28}$$

subject to

$$
\begin{aligned}
& x_t \geq 0, \\
& z_{t+1} = \min\{\overline{z}, \underline{z} + \phi x_t\}, \\
& c_t^R = \tau_t z_t \widetilde{Y}_t - \eta_A A_{t+1}^{\sigma_A} - \eta_P P_{t+1}^{\sigma_P} - x_t, \\
& \widetilde{Y}_{t+1} = P_{t+1}(BA_{t+1})^{\alpha} L_{t+1}^{1-\alpha}, \\
& L_{t+1} = \gamma(1-\tau_t)\widetilde{Y}_t.
\end{aligned} \tag{A.29}
$$

(We could also add the constraint that $P_{t+1} \leq 1$, but we here assume that the exogenous parameter values are such that this constraint never binds, which can be assured by setting η_P sufficiently large.)

The task undertaken here is to show that the maximization problem in (A.28) and (A.29) can be rewritten as that in (10) and (11) in the paper (which refers to the benchmark model), but with η and σ being functions of "deep" parameters, such as η_A, σ_A, η_P, and σ_P.

Maximizing (A.28) subject to (A.29), the first-order conditions with respect to P_{t+1} and A_{t+1} (in an interior solution) are given by

$$
\begin{aligned}
(1-\beta)\left[c_t^R\right]^{-1} \sigma_P \eta_P P_{t+1}^{\sigma_P - 1} &= \beta P_{t+1}^{-1}, \\
(1-\beta)\left[c_t^R\right]^{-1} \sigma_A \eta_A A_{t+1}^{\sigma_A - 1} &= \alpha\beta A_{t+1}^{-1}.
\end{aligned} \tag{A.30}
$$

Dividing these two first-order conditions with each other, and rearranging, we can write the optimal spending by the ruler on defense as a constant times optimal spending on other public goods:

$$
\eta_P P_{t+1}^{\sigma_P} = \frac{1}{\alpha}\left(\frac{\sigma_A}{\sigma_P}\right)\eta_A A_{t+1}^{\sigma_A}, \tag{A.31}
$$

which in turn allows us to write total spending on defense and public goods as

$$
\eta_P P_{t+1}^{\sigma_P} + \eta_A A_{t+1}^{\sigma_A} = \left[\frac{\sigma_A + \alpha\sigma_P}{\alpha\sigma_P}\right]\eta_A A_{t+1}^{\sigma_A}. \tag{A.32}
$$

Next we can solve (A.31) for P_{t+1} to write

$$
P_{t+1} = \left[\frac{1}{\alpha}\left(\frac{\sigma_A}{\sigma_P}\right)\left(\frac{\eta_A}{\eta_P}\right)\right]^{\frac{1}{\sigma_P}} A_{t+1}^{\frac{\sigma_A}{\sigma_P}}. \tag{A.33}
$$

From (A.33) we note that

$$
P_{t+1}^{\frac{1}{\alpha}} A_{t+1} = \left[\frac{1}{\alpha}\left(\frac{\sigma_A}{\sigma_P}\right)\left(\frac{\eta_A}{\eta_P}\right)\right]^{\frac{1}{\alpha\sigma_P}} \widetilde{A}_{t+1}, \tag{A.34}
$$

where we define

$$
\widetilde{A}_{t+1} = A_{t+1}^{\frac{\sigma_A + \alpha\sigma_P}{\alpha\sigma_P}}. \tag{A.35}
$$

We can now use (A.34) to write (A.25) as

$$\widetilde{Y}_{t+1} = P_{t+1}(BA_{t+1})^{\alpha} L_{t+1}^{1-\alpha} = \left(BP_{t+1}^{\frac{1}{\alpha}} A_{t+1} \right)^{\alpha} L_{t+1}^{1-\alpha} = \left(\widetilde{B} \widetilde{A}_{t+1} \right)^{\alpha} L_{t+1}^{1-\alpha}, \tag{A.36}$$

where

$$\widetilde{B} = B \left[\frac{1}{\alpha} \left(\frac{\sigma_A}{\sigma_P} \right) \left(\frac{\eta_A}{\eta_P} \right) \right]^{\frac{1}{\alpha \sigma_P}}. \tag{A.37}$$

Similarly, using (A.32), and the definition of $\widetilde{A}_{t+1}$ in (A.35), we can write total spending on public goods (including defense) as

$$\eta_P P_{t+1}^{\sigma_P} + \eta_A A_{t+1}^{\sigma_A} = \left[\frac{\sigma_A + \alpha \sigma_P}{\alpha \sigma_P} \right] \eta_A \left(\widetilde{A}_{t+1} \right)^{\frac{\alpha \sigma_P \sigma_A}{\sigma_A + \alpha \sigma_P}}. \tag{A.38}$$

Next we can define

$$\begin{aligned} \eta &= \left[\tfrac{\sigma_A + \alpha \sigma_P}{\alpha \sigma_P} \right] \eta_A, \\ \sigma &= \tfrac{\alpha \sigma_P \sigma_A}{\sigma_A + \alpha \sigma_P}, \end{aligned} \tag{A.39}$$

to write (A.38) as

$$\eta_P P_{t+1}^{\sigma_P} + \eta_A A_{t+1}^{\sigma_A} = \eta \widetilde{A}_{t+1}^{\sigma}. \tag{A.40}$$

This allows use to rewrite (A.29) as

$$\begin{aligned} & z_{t+1} = \min\{\overline{z}, \underline{z} + \phi x_t\}, \\ & x_t \geq 0, \\ & c_t^R = \tau_t z_t \widetilde{Y}_t - \eta \widetilde{A}_{t+1}^{\sigma} - x_t, \\ & \widetilde{Y}_{t+1} = \left(\widetilde{B} \widetilde{A}_{t+1} \right)^{\alpha} L_{t+1}^{1-\alpha}, \\ & L_{t+1} = \gamma (1 - \tau_t) \widetilde{Y}_t, \end{aligned} \tag{A.41}$$

where the only changes are to the third and fourth rows, here rewritten by using (A.36) and (A.40).

Maximizing (A.28) subject to (A.41) amounts to the exact same maximization problem as in the benchmark model; see (10) and (11). The only difference is that A_t, B, Y_t, and Y_{t+1} are replaced by the corresponding "tilde" variables: $\widetilde{A}_{t+1}$, $\widetilde{B}$, $\widetilde{Y}_t$, and $\widetilde{Y}_{t+1}$, respectively.

Note that a territory that is easier to protect, which can be interpreted as η_P being low, is associated with a high $\widetilde{B}$, while η and σ do not depend on η_P; see (A.37) and (A.39). Intuitively, when defense is less expensive, fewer resources are needed for investment in defense. These can be invested in productive capacity instead, which translates to more output holding total expenditures constant.

Incomes of ruler and subjects in the low-extractive steady state

In the low-extractive steady state, after-tax income per subject equals

$$(1-\underline{\tau})\frac{\underline{Y}}{\underline{L}}=\frac{1}{\gamma}, \tag{A.42}$$

which can be derived by imposing steady state on (5). That is, per-subject income after tax only depends on the reproduction parameter, γ, as in any Malthusian steady state. In particular, it is independent of $\underline{z}$.

Next we derive an expression for the ruler's income in the same low-extractive steady state. First note that the number of subjects is technically infinite (i.e., a continuum of agents of mass $\underline{L}$). To make our income comparisons mathematically consistent, below we interpret the ruler as a collective of constant mass one, although we still refer to him in the singular.

Recall that the ruler's income in the low-extrative steady state equals $\underline{z\tau Y}$ (divided by a mass of 1). From (17) we see that

$$\begin{aligned}\underline{Y} &= \left[\kappa D B^{\alpha} \underline{z}^{\alpha-1}\left(\phi \underline{z}\right)^{\rho}\right]^{\frac{1}{1-\rho}}, \\ &= \left[\kappa D B^{\alpha}\phi^{\rho}\right]^{\frac{1}{1-\rho}} \underline{z}^{\frac{\alpha-1+\rho}{1-\rho}} \\ &= \left[\kappa D B^{\alpha}\phi^{\rho}\right]^{\frac{1}{1-\rho}} \underline{z}^{\frac{\alpha-(1-\rho)}{1-\rho}} \\ &= \left[\kappa D B^{\alpha}\phi^{\rho}\right]^{\frac{1}{1-\rho}} \underline{z}^{\frac{\alpha}{1-\rho}-1}.\end{aligned} \tag{A.43}$$

On closer inspection of the last equality in (A.43), recalling from (46) that $\rho = 1-\alpha+\alpha/\sigma$, the first term in the exponent on $\underline{z}$ can be written

$$\frac{\alpha}{1-\rho}=\frac{\alpha}{1-[1-\alpha+\alpha/\sigma]}=\frac{\sigma}{\sigma-1}>0, \tag{A.44}$$

where the inequality follows from $\sigma>1$. Now (A.43) and (A.44) show that the ruler's income can be written

$$\underline{z\tau Y}=\underline{\tau}\left[\kappa D B^{\alpha}\phi^{\rho}\right]^{\frac{1}{1-\rho}} \underline{z}^{\frac{\sigma}{\sigma-1}}. \tag{A.45}$$

Recall that $\underline{\tau}$ is given by (14), and that κ and D are given by (47) and (54). These all depend on exogenous parameters, but not on $\underline{z}$. It follows that $\underline{z\tau Y}$ is strictly decreasing in $\underline{z}$, and that

$$\lim_{\underline{z}\to 0}\underline{z\tau Y}=0.$$

That is, the ruler's income in the low-extractive steady-state, $\underline{z\tau Y}$, can be made arbitrarily small by setting $\underline{z}$ arbitrarily close to zero. Specifically, it can be made smaller than (or equal to) after-tax income per subject, which is given by (A.42) and indendent of $\underline{z}$.
